# The Cooperation Databank: Machine-Readable Science Accelerates Research Synthesis

**DOI:** 10.1177/17456916211053319

**Published:** 2022-05-17

**Authors:** Giuliana Spadaro, Ilaria Tiddi, Simon Columbus, Shuxian Jin, Annette ten Teije, CoDa Team, Daniel Balliet

**Affiliations:** 1 Department of Experimental and Applied Psychology, Vrije Universiteit Amsterdam; 2 Institute for Brain and Behavior Amsterdam (IBBA), Vrije Universiteit Amsterdam; 3 Department of Computer Science, Vrije Universiteit Amsterdam; 4 Department of Psychology, University of Copenhagen

**Keywords:** cooperation, social dilemmas, databank, meta-analysis, knowledge representation, ontologies

## Abstract

Publishing studies using standardized, machine-readable formats will enable machines to perform meta-analyses on demand. To build a semantically enhanced technology that embodies these functions, we developed the Cooperation Databank (CoDa)—a databank that contains 2,636 studies on human cooperation (1958–2017) conducted in 78 societies involving 356,283 participants. Experts annotated these studies along 312 variables, including the quantitative results (13,959 effects). We designed an ontology that defines and relates concepts in cooperation research and that can represent the relationships between results of correlational and experimental studies. We have created a research platform that, given the data set, enables users to retrieve studies that test the relation of variables with cooperation, visualize these study results, and perform (a) meta-analyses, (b) metaregressions, (c) estimates of publication bias, and (d) statistical power analyses for future studies. We leveraged the data set with visualization tools that allow users to explore the ontology of concepts in cooperation research and to plot a citation network of the history of studies. CoDa offers a vision of how publishing studies in a machine-readable format can establish institutions and tools that improve scientific practices and knowledge.

Scientists struggle to keep pace with the literature and to efficiently make comparisons between the results of different studies. These challenges are not getting any easier, given an ever-increasing number of publications per year (Bornmann & Mutz, 2015) and a growing number of scientists facing increasing demands to publish (Grimes et al., 2018; Van Dalen & Henkens, 2012). This expansion of the literature is co-occurring with an awareness that research is not always reproducible and replicable (Baker, 2016; Open Science Collaboration, 2015), which further stresses the importance of comparing study results and surveying the entire body of evidence (Gurevitch et al., 2018; Murad & Montori, 2013). Nonetheless, searching and comparing results across studies is a difficult and time-consuming enterprise.

Part of the problem is that scientists report their study results online in PDFs and data sets, which tend to be unstandardized and not machine readable. Therefore, an expert must diligently search, select, annotate, and compare results across PDFs and data sets—the domain of research synthesis and meta-analysis. However, meta-analysis is often inaccessible to all but experts who have been trained in these methods. In addition, a meta-analytic project can take many years to complete, and by the time the meta-analysis is eventually published, some of the existing data may already be outdated (Beller et al., 2013).

In recent years, knowledge-representation methods developed in the field of artificial intelligence have offered key solutions to these challenges (Seringhaus & Gerstein, 2007; Shotton et al., 2009). These methods can be applied to represent information from studies in a structured, meaningful way (i.e., semantically enhanced data), which is also machine readable. This could enable search engines to reason efficiently over a structured representation of studies and their results (e.g., effect sizes) and to effectively obtain comparable information that can be used to conduct on-demand meta-analyses. Indeed, several data-curation projects in the social and behavioral sciences have begun to apply these techniques to realize these benefits (Bergmann et al., 2018; Bosco et al., 2015).

We have applied methods of knowledge representation and built on these previous efforts to develop the Cooperation Databank (CoDa), which is (a) a semantically enriched data set of correlational and experimental studies on human cooperation and (b) a research platform that can perform several functions, including on-demand meta-analyses of these studies, estimates of publication bias in the literature, and statistical power analyses for future studies. Here, we describe the ontology we developed to represent the concepts and study results in this literature and discuss the functional benefits enabled by this approach through a description of the CoDa research platform. We structure the article according to the process used to create CoDa (see Fig. 1), which can guide efforts to produce similar resources for other topics in the social and behavioral sciences.

**Fig. 1.**
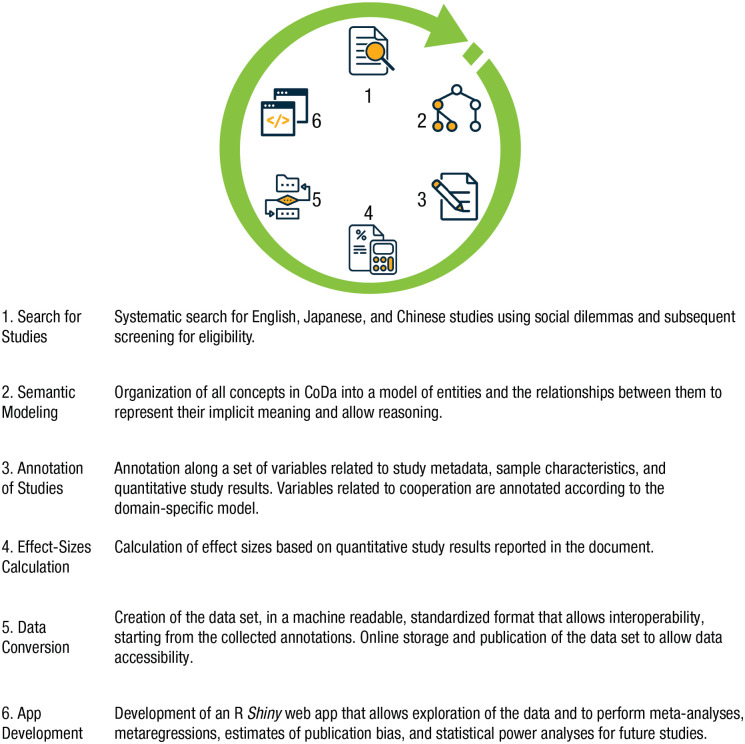
The process of developing the Cooperation Databank (CoDa). The steps were performed in the presented order, but there was some feedback and iterations that occurred between specific steps. For example, some changes to the semantic modeling were made using knowledge acquired during the annotation of studies.

## Human Cooperation in Social Dilemmas

CoDa contains the history of empirical research on human cooperation using social dilemmas. Social dilemmas have received intensive interdisciplinary attention since the 1950s and rose to prominence in both theoretical and experimental research on cooperation following the inception of game theory (Camerer, 2011; Deutsch, 1958; Pruitt & Kimmel, 1977; Van Lange et al., 2014; Von Neumann & Morgenstern, 1944). In a social dilemma, each person can achieve the best outcome by choosing to defect and exploiting a cooperative partner, but if all persons defect, then each person obtains a worse outcome than when all cooperate. The worst possible outcome in such situations results from deciding to cooperate but then being exploited by a noncooperative partner. This type of situation is captured by the prisoner’s dilemma but also by decisions about costly contributions to public goods or sharing of common pool resources. Indeed, many contemporary societal issues can be understood as social dilemmas, including tax evasion, resource conservation, and climate change (Milinski et al., 2006; Yoeli et al., 2013).

In the mid-1950s, an experimental-research tradition emerged that used highly standardized social-dilemma paradigms, such as the prisoner’s dilemma, as a framework to study cooperative behavior (see Deutsch, 1949, 1958; Rapoport & Chammah, 1965). In these studies, individuals make decisions under a specified interdependent payoff structure (see Box 1), and variation in cooperative behavior is examined in relation to variation in features of the situation or individual differences (e.g., personality). This research tradition continues today and is represented across numerous disciplines in the social and biological sciences (for reviews, see Chaudhuri, 2016; Dawes, 1980; Rand & Nowak, 2013; van Dijk & De Dreu, 2021; Van Lange et al., 2014).

**Box 1**

Social-dilemma paradigms included in the Cooperation Databank. In the prisoner’s dilemma (a), participants decide independently whether to cooperate (transfer any portion of their endowment to the partner) or defect. The transfer is multiplied by a constant *m* (*m* > 1) and added to the partner’s endowment. In the public-goods dilemma (b), each member of a group of size *N* decides how much of an individual endowment to contribute to a group account. Contributions are multiplied by a constant *m* (1 < *m* < *N*) and shared equally across all group members, irrespective of their individual contributions. In the resource dilemma (c), each member of a group of size *N* decides how much to take from a common resource. The amount each member takes is no longer available to other group members. After each round, the resource can recover with reproduction rate *r* > 1. The game ends once the resource is depleted.

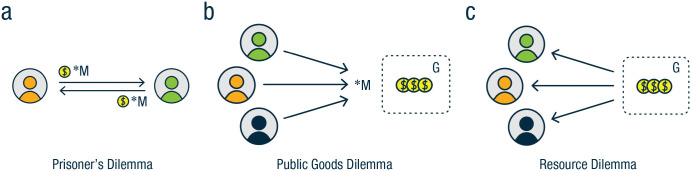

## Development of CoDa

### Search for studies

To create a machine-readable databank of studies of human cooperation, we applied standardized methods for searching for experimental studies of human cooperation. We conducted an extensive systematic search for studies published until 2017 in English, Chinese, or Japanese as articles, book chapters, working papers, or doctoral dissertations. We included studies with human participants interacting in a situation containing a conflict of interests with a structure of possible outcomes modeled as a prisoner’s dilemma, public-goods game, or resource dilemma (see Box 1). All studies had to report the mean levels of cooperative behavior observed in the social dilemma or enough other quantitative information to allow for the computation of standardized effect sizes. More details about the search strategy are provided in the Supplemental Material available online.

**Fig. 2.**
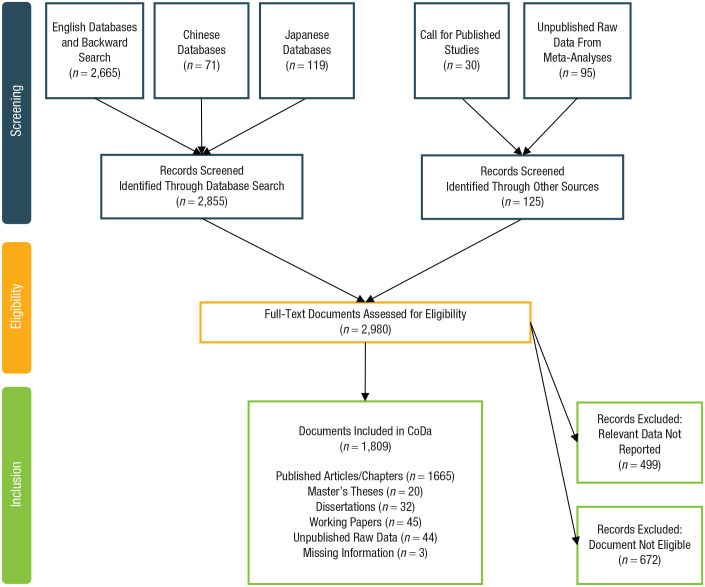
Flow chart of literature search and inclusion of studies in the Cooperation Databank.

As a result, CoDa includes a total of 2,636 studies extracted from 1,809 documents published between 1958 and 2017 conducted in 78 societies and contains a total of 356,283 participants. Figure 2 displays a flow diagram detailing the outcome of our search for studies. Next, we modeled the information and concepts contained in studies of human cooperation in an ontology that could be used to annotate studies and developed a semantically enhanced research platform to explore the databank, effectively select studies to be included in a meta-analysis, and ultimately enable the performance of meta-analyses on demand.

### Semantic modeling: an ontology of research on human cooperation

An ontology is a conceptualization of a domain as a body of formally represented knowledge in the form of concepts that are assumed to exist in an area of interest and the relationships that hold among them (Gruber, 1995). It is represented in the form of graphs of nodes and edges, in which the nodes correspond to concepts of the domain, called *classes*, and the edges correspond to attributes of a concept or relationships between concepts and are called *properties*. When also including individuals or *instances* belonging to the classes, an ontology is often referred to as a knowledge graph. Ontologies are *explicit* (i.e., expressed in the form of machine-readable formats such as RDF [RDF Working Group, 2014a], RDF(S) [RDF Working Group, 2014b], and OWL [OWL Working Group, 2012]) in a way that they can be *shared* (i.e., software agents can publish, exchange, and reuse such information using a common language consisting of standard web protocols HTTP [IETF httpbis Working Group, 2014] and URI [URI Planning Interest Group, 2001]). In other words, ontologies are a means to automatically share knowledge using a content-specific language.

Although previous reviews of cooperation research have attempted to categorize different research topics and findings (Parks et al., 2013; Weber et al., 2004), to the best of our knowledge, no ontology exists to represent this body of knowledge. We therefore developed the Ontology of Human Cooperation Studies, which consists of a graph of entities and relationships describing both the domain-independent bibliographic information and the domain-specific knowledge from the collected studies. We used an ontology engineering approach (Noy & McGuinness, 2001) that consists of (a) defining classes in the ontology (i.e., concepts in the domain of human cooperation), (b) arranging the classes in a taxonomy (i.e., a hierarchy of subclasses and superclasses), (c) defining the relationships between the classes, and (d) defining instances belonging to each class and the relationships between them.

#### CoDa’s domain-independent model

**Fig. 3.**
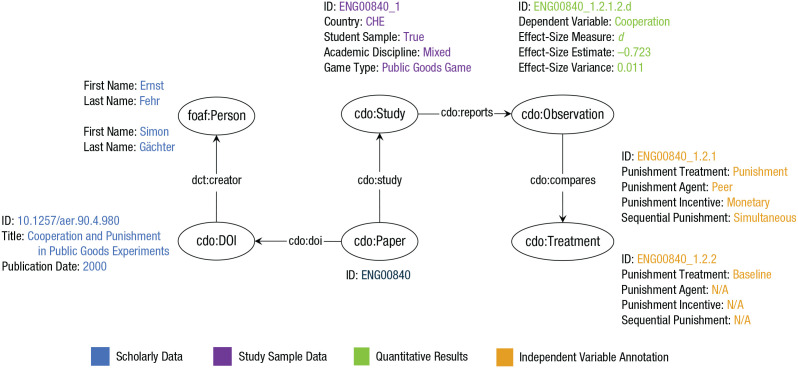
Domain-independent and domain-specific model of the Cooperation Databank (CoDa) applied to one annotated instance. foaf:Person, cdo:DOI, cdo:Paper, and cdo:Study are part of the domain-independent model in the CoDa knowledge graph. Cdo:Treatment and cdo:Observation are part of the domain-specific model. However, some variables included in cdo:Study (e.g., game type) are pertinent only to represent cooperation studies. The figure displays a small selection of variables on which the study was annotated. For the complete list of variables for which studies were annotated, see the Supplemental Material available online.

We first established a general ontological schema for empirical research studies by using all variables coded for an article as metadata (e.g., DOIs and authors), studies (e.g., sample characteristics and study characteristics), and observations (e.g., the relation between an independent variable and dependent variable), as in Figure 3.

In the CoDa knowledge graph, each publication is represented as a cdo:Paper that includes an arbitrary set of cdo:Study; each study reports one or more effect-size estimates that we model as cdo:Observation and that is in turn modeled as involving one or two cdo:Treatment.^2^ Each cdo:Paper also has a DOI identifier, represented by the cdo:DOI class; additional metadata, such as publication date and publication status, are provided as properties of this class. Furthermore, additional specific properties are included as attributes of the class cdo:Study, respectively representing information about the study sample (e.g., the total sample size, cdo:overnallN) or quantitative/statistical information (e.g., the overall cooperation rate, cdo:overallProportionCooperation).

#### CoDa’s domain-specific model

**Fig. 4.**
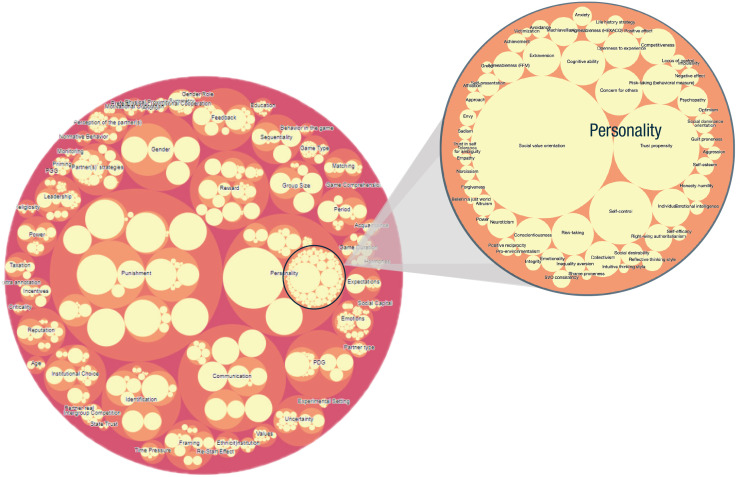
Packed bubble chart of the variables used to predict cooperation and for which meta-analyses are possible using the Cooperation Databank (CoDa) with a zoom-in of personality variables. This image displays the Ontology Explorer tool on the CoDa application for users to navigate the variables. The relative size of each bubble represents the relative number of treatments that was annotated with that variable.

We then organized all variables annotated at treatment level in a fine-grained, domain-specific schema representing all classes and relationships related to cooperation in social dilemmas. The organization into classes was achieved using a bottom-up approach; all treatment-level coded variables were the most specific classes (i.e., the leaves of the hierarchy), and we subsequently grouped them into more general concepts. The resulting hierarchy is shown in Figure 4 and includes independent variables, such as (a) variables related to the participants in the study, including personal background (age, ethnicity, education), personality traits (e.g., HEXACO, trust), and dynamic psychological states (e.g., emotions, partner evaluations); (b) variables related to the structural aspects of the game, such as participant payment, degree of conflicting interests, and repetition of interactions; (c) variables related to the decision context and that do not affect the game structure, such as intrapersonal features (e.g., priming) or interpersonal features (e.g., gossip); and (d) variables related to rules and norms for participants in the game, such as punishment, reward, or taxation.

To simplify the ontology readability, we made the last layer in the hierarchy correspond to the 233 coded variables at treatment level. All variables and their definition are reported in the Codebook on OSF (https://osf.io/mdcp6/).

### Annotation of studies

We then annotated the information contained in the documents into a machine-readable, standardized format. For each study, we annotated characteristics of the study sample and protocol, including characteristics of the continuous variables and specific treatments that were associated with cooperation in the studies. These characteristics can be classified into five categories: (a) scholarly properties (e.g., the DOI, or in the absence thereof, author names, journal information, etc.); (b) sample properties (e.g., sample size, mean age, nationality, percentage male); (c) study properties (e.g., group size, number of iterations, and degree of conflicting interests); (d) quantitative properties (e.g., mean level and standard deviation of cooperation in each treatment of a study), including the statistical information that quantifies the relation between a (measured or manipulated) variable and cooperation (e.g., means, test statistics), which we used to compute standardized effect sizes—quantified information about the direction and magnitude of an association between observed or manipulated variables and cooperation; and (e) any (observed or manipulated) variable that was related to cooperation (e.g., a personality trait, type of punishment, or expectations of other’s cooperation). We annotated information about the relationship each variable had with cooperation (i.e., the dependent variable) and separately described each treatment. For every treatment, the annotation contains information about the variable that was observed or manipulated in a specific study. To illustrate, if a study manipulated the presence versus absence of punishment across two treatments, we annotated each treatment as containing (vs. not containing) a punishment mechanism. We also annotated variations across different punishment treatments (e.g., who enacted the punishment, the cost-effectiveness of punishment, and type of rule used to mete out punishment). The Codebook provides an overview of all the possible variables annotated for each study, including the operational definitions and their levels of annotation.

We took several steps to reduce human error and estimate the interrater agreement in the annotation of studies. First, we followed an intensive protocol to train domain experts to annotate studies (for details, see the Supplemental Material). Second, two domain experts independently annotated 100% of the mean values and standard deviations for each study (and each treatment per study), which we use as a primary means to calculate effect sizes. Third, we estimated the error in each variable by reannotating a random selection of 10% of the articles. Most variables had interrater agreement of Krippendorff’s α above .70, a criterion often used for publishing in the social sciences (for details, see the Supplemental Material). That said, a few variables did fall below this threshold (e.g., number of trials, number of blocks), and so these variables were removed from CoDa.

### Effect-size calculations

Standardized effect-size estimates are the input to meta-analyses and represent the relation between a variable and cooperation. Effect sizes can take different forms, such as the correlation coefficient (to quantify the association between two continuous variables) or the standardized mean difference (to quantify the difference between two levels of a categorical variable in terms of a continuous outcome variable). However, effect sizes are often unreported, and we used the annotated quantitative information (e.g., correlation coefficients, means, standard deviations, and test statistics) to calculate all possible effect-size estimates. We computed standardized effect-size estimates for the effects of continuous and categorical variables. For categorical variables, we computed an effect-size estimate for each combination of two treatments used to manipulate a variable within a study. Effect-size computations were implemented using the package *esc* (Lüdecke, 2019) for the R software environment (R Core Team, 2022).^1^ Because studies report a variety of different information, we computed estimates of two measures—the correlation coefficient *r* and Cohen’s *d*—using multiple algorithms. For example, Cohen’s *d* for a two-group comparison with a continuous outcome can be computed from means, standard deviations, and sample sizes but also from the *t* statistic or be approximated from the *p* value. Whenever possible, we also provide conversions between *r* and *d* to allow meta-analyses across a wide range of designs. Overall, 7,631 effect-size estimates could be computed using the annotated quantitative information. Further details on the how effect sizes are calculated are provided in the Supplemental Material.

### Data conversion and publication

We used the ontology and annotated studies to create a CoDa knowledge graph guided by the principles of findability, accessibility, interoperability, and reusability of data (FAIR; Wilkinson et al., 2016). To do so, we converted the coded tabular data into a structured, graph-shaped data set that is published openly and can be queried on demand. To achieve this, we followed the steps below:

1. We described CoDa with rich machine-readable metadata that allow the data set to be indexed and found automatically (principle of findability).
2. We converted every data item in a web-friendly standard (i.e., a URL that allows data discovery on the web; principle of accessibility).
3. We stored the data in formats that are standardized by the community and link these data to other existing data sets (principle of interoperability).
4. We allowed the data to be publicly accessible, replicable, used by others in novel ways (principle of reusability).

The generated data set, summarized in Table S3 in the Supplemental Material, is hosted in a TriplyDB graph database and can be accessed at https://data.cooperationdatabank.org/. Whenever possible, we have aligned CoDa with existing data sets, for example, MeSH (Sewell, 1964), Wikidata (Vrandecˇić & Krötzsch, 2014) and ORCID (Haak et al., 2012), to encourage reusability (for details, see the Supplemental Material).

One of the major benefits of storing the scientific information in this format is the possibility to implement and publish living reviews—up-to-date summaries of research findings that are automatically updated as new information becomes available (Elliott et al., 2014). The implementation and publication of living reviews is an emerging approach to research synthesis and is particularly promising for research fields in which evidence is growing at a fast pace and has implications for resources allocations (Elliott et al., 2017). As proof of concept, we generated a living review that is published online and provides overall descriptive summary of the annotated data in CoDa, which users can query and customize on demand (https://cooperationdatabank.org/data-stories/whats-in-the-databank/).

### Community-augmented meta-analytic systems and the CoDa knowledge graph

Ontologies and knowledge graphs can advance community-augmented meta-analytic (CAMA) systems. CAMA projects are standardized collections of research findings stored in repositories that can be summarized through meta-analytic tools (Burgard et al., 2021; Tsuji et al., 2014). CoDa shares several goals with three other curation projects within the psychological sciences and complements these existing projects (for a comparative overview of existing CAMA systems, see Burgard et al., 2021). MetaLab is a platform that supports collaborative hosting and curation of existing published meta-analyses of studies in the domain of language acquisition and cognitive development (Bergmann et al., 2018). Through its dynamic interface, users can visualize meta-analytic results and perform power analyses and simulations under a variety of conditions. PsychOpen CAMA is under development and is intended to contain data from single meta-analyses across domains within psychology (Burgard et al., 2021). In contrast to these two projects, CoDa includes all existing studies conducted within an entire field of study, has a corresponding ontology that formally represents this knowledge in a machine-readable way, and allows queries and analyses across subdomains.

MetaBUS is a databank of findings in the field of applied psychology and includes a broad, standardized taxonomy that enables both enhanced literature search and targeted and exploratory meta-analyses (Bosco et al., 2015). MetaBUS uses a semiautomated process of extracting quantitative information from correlation matrices reported in articles, which are then manually classified according to the taxonomy and along a set of descriptors. The use of correlation matrices is a standard practice to report findings in the field of applied psychology and accounts for the majority of the reported findings (Aguinis et al., 2011). However, this is not the case for other fields (e.g., human cooperation), in which findings are commonly reported as mean differences across treatments. The CoDa knowledge graph represents the mean levels of cooperation per treatment and so can accommodate research findings from both correlational and experimental methods. Moreover, a unique feature of the CoDa research platform allows users to perform on-demand cross-cultural analyses by nesting effects within countries/regions and including moderators at the country level (e.g., population demographics, economy, and cultural values). These open-access cross-societal indices are hosted in the platform and can be directly related to the outcome of studies to evaluate how cooperation varies across societies.

Ontologies promise to integrate, communicate, and ultimately, advance scientific knowledge. This promise is already being realized in several disciplines. For example, ontologies are being used in the biosciences to facilitate research synthesis. The Gene Ontology (Ashburner et al., 2000), for instance, summarizes findings from almost 163,000 scientific publications about gene functions and has become an institution for data-driven genomic science. An ontology-based approach has also been adopted in the field of behavioral change, in which the annotation of the characteristics and context of behavioral interventions, together with an evaluation component, allows researchers and practitioners to answer key questions about interventions (Michie et al., 2017, 2021). Applying an ontology-based approach to any domain of empirical knowledge provides two main benefits (Norris et al., 2019). First, ontologies integrate different theoretical frameworks and facilitate the comparison and sharing of concepts across scientific disciplines. Second, ontologies allow an abundance of research to be synthesized efficiently, which provides several benefits, including conducting on-demand meta-analyses. Whereas the former benefit is achievable only if several disciplines adopt a similar approach, the latter benefits of ontologies can be rapidly realized. Next, we describe the functions enabled by the CoDa research platform, which demonstrate how this approach can benefit and accelerate psychological and behavioral science.

### The CoDa research application and platform

We used our Ontology of Human Cooperation Studies to develop a semantically enhanced research platform to explore the databank, effectively select studies to be included in a meta-analysis, and ultimately enable the performance of meta-analyses on demand. The CoDa research platform enables users to search and select data from the CoDa knowledge graph and perform analyses on the sample of studies from the search result. The research platform is built as a web-based interface using R *Shiny* (Chang et al., 2020), which has been used to build the interactive dashboards of metaBUS and MetaLab to perform similar on-demand computations. The main activities of the platform are described in the rest of this section and summarized in Table 1. Further information intended for users (e.g., video tutorials on how to perform various activities on the platform) is accessible at https://cooperationdatabank.org/.

**Table 1.** List of Activities and Tasks That Can Be Performed by Users of the Cooperation Databank Platform

Activity	Tasks
Search	• Tabular exploration • Full data access via TriplyDB (querying by Elasticsearch and SPARQL)
Selection	• Filtering studies based on: - study characteristics - sample characteristics - article metadata - quantitative results - independent variables • Individualized addition and exclusion of studies
Meta-analyses	• Synthesize the effect size of variables related to cooperation • Choice of: - type of effect size (*r* or *d*) - model (e.g., random vs. fixed effects; estimator) • Cluster effect sizes within articles, studies, and countries/regions • Selection of moderators at different levels (specific to the variable, study/sample characteristics, and country/region) to predict the effect size
Metaregression	• Predict (logit-transformed) mean cooperation rates • Choice of: - model (e.g., random vs. fixed effects; estimator) • Clustering logit-transformed mean cooperation within papers, studies, and countries/regions • Selection of moderators at different levels (specific to the variable, study/sample characteristics, country/region) to predict the logit of mean cooperation rates
Publication bias	• Assessment of and adjustment for publication bias (i.e., trim-and-fill method, rank correlation test, Egger’s regression test, Henmi-Copas method)
Power analysis	• Selection of: - test family (e.g., *t* test, correlation) - test type (e.g., two- vs. one-sample *t* test) • Definition of the level of α (significance) and β (power) to detect the effect size
Visualizations	• Summary of selection by year of data collection, country/region, and sample size • Summary of meta-analysis using a forest plot, violin plot, funnel plot • Data table of each study and their effect size and moderator values • Visualization of citation networks • Visualization of the ontology
Crowdsourcing	• Ad hoc inclusion of additional effects while performing a meta-analysis or metaregression
Download	• Download of selected data • Download of references formatted in American Psychological Association style and machine readable • Download of citation communities

#### Search and selection of studies

Users can conduct a faceted search using the CoDa schema described above to select observations that contain specific variables, such as personality, communication, and framing (Box 2b). Selected studies are visualized according to the country/region where the studies were conducted, sample size per study, and year the study was conducted (Box 2a). The selection of observations can be refined using more specific criteria, such as the type of personality trait (e.g., any of the 60 traits in the databank), communication occurrence (e.g., one-shot or ongoing), and framing manipulation (e.g., gain or loss). Users can combine up to six such selections to define each treatment to be included in the meta-analysis. This allows users to conduct meta-analyses on any effect for which data are available.

**Box 2**

The overview of the studies in (a) displays the number of effect sizes, articles, studies, and participants that is the output of a search and selection of studies; studies can be displayed according to country/region of participants, year the study was conducted, and sample size. The selection of studies in (b) displays the search and selection tool through which users can identify and curate a set of studies manipulating framing (gain vs. loss) to be used for meta-analysis.

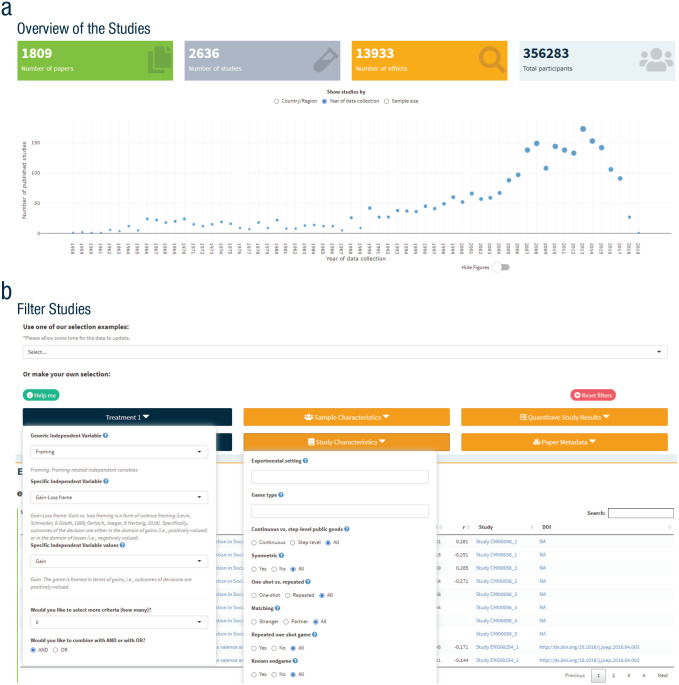

The platform also allows users to select studies that meet specific inclusion criteria, such as being conducted only within a specific country/region during a specific range of years. These options can help users navigate the existing literature and identify studies that are highly comparable to be included in a meta-analysis. Finally, users can also personalize the meta-analysis by removing or adding individual studies. The selection of studies and their study characteristics are displayed in a table and can be easily downloaded and used in analyses while working offline (see Fig. S1 in the Supplemental Material). Users can also obtain a list of references for the selected studies.

#### Analyses of selected studies

After the user has selected observations to be included, a meta-analysis can be run under the Meta-Analyses tab. Meta-analyses are conducted on effect sizes for cdo:Observations, which express either a relationship between a continuous variable and cooperation or the difference in cooperation between two treatments. The analyses are run using the R package *metafor* (Viechtbauer, 2010). CoDa implements many of the state-of-the-art functionalities of *metafor*, including meta-analyses of effect sizes, metaregressions of (logit-transformed) mean cooperation rates, flexible inclusion of moderators to predict effect sizes (and mean cooperation rates), and multilevel meta-analyses. For a standard meta-analysis, users can select (a) the effect size used in the meta-analysis (i.e., *r*, *d*), (b) random- versus fixed-effects models, and (c) estimators of residual heterogeneity. The output is displayed in a table formatted similarly to published articles (see Fig. S2 in the Supplemental Material), but the raw R output can also be obtained. Meta-analyses can also be conducted on standardized mean levels of cooperation observed across studies and/or treatments (e.g., Engel, 2011). Finally, the user may also conduct multilevel meta-analysis to account for dependencies in the data. CoDa enables the specification of (crossed) random factors for study, article, and country/region, and when users select cluster errors according to both articles and studies, then studies are nested within articles. The outputs of all analyses are displayed in interactive visualizations, including forest plots (see Box 3a) and violin plots (see Fig. S3 in the Supplemental Material).

**Box 3**

The forest plot in (a) displays each study and its corresponding effect size and confidence interval. The funnel plot in (b) displays studies according to their effect size and standard error and can be used to evaluate publication bias.

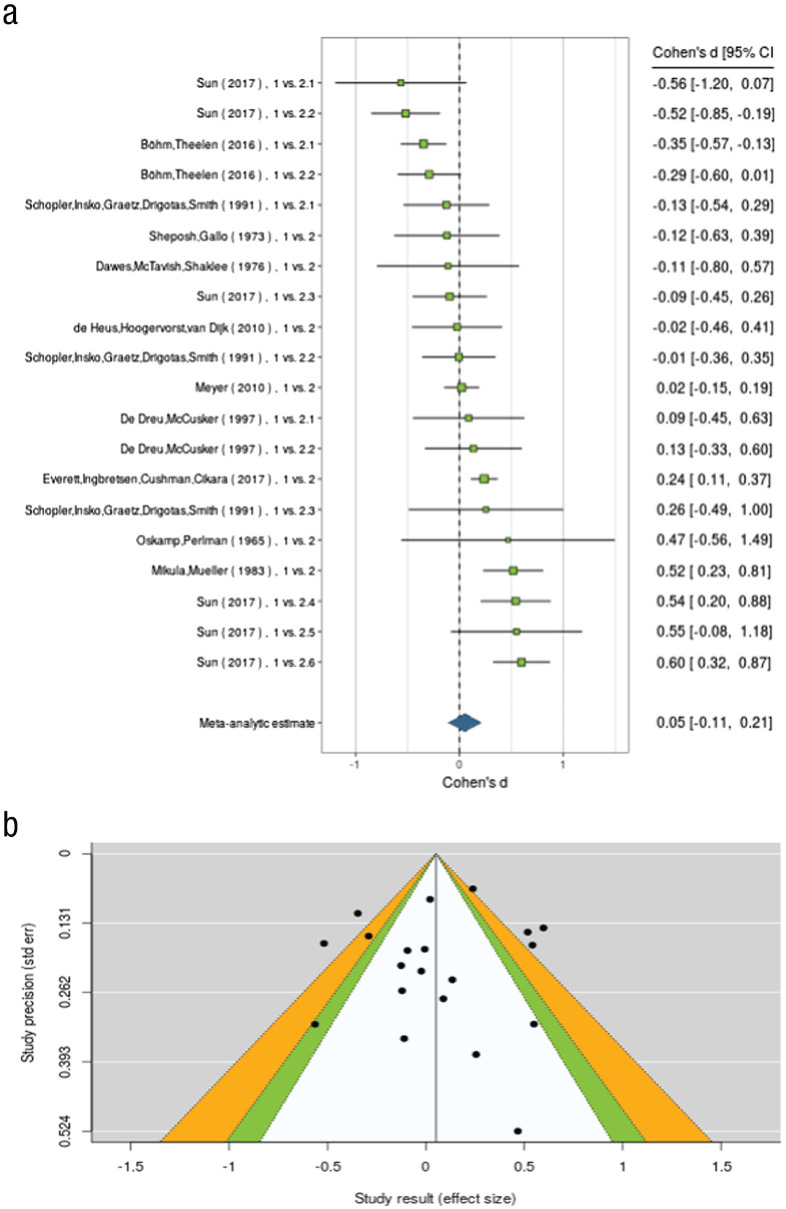

The user may also select any number of moderators to be included in a multivariate model predicting the effect sizes. Moderator variables can be chosen at the observation level (“variable moderators”), the study level (“study moderators”), and country level (“country/region moderators”). For example, when doing a meta-analysis on the effect of rewards on cooperation, the user could select (a) a variable moderator, which indicates a specific way in which rewards were implemented (e.g., the rewarding agent); (b) a study-level moderator (e.g., group size); and (c) a country-level moderator (e.g., rule of law). Note that the platform contains a large number of cross-societal indices (e.g., gross domestic product, trust, and government effectiveness) that can be used to analyze how the results of studies vary across societies and cultures. The Supplemental Material contains additional details about how these analyses are implemented in CoDa.

#### Publication-bias analyses

A central challenge to meta-analyses is that many literatures suffer from publication bias (Ferguson & Heene, 2012; Van Aert et al., 2019). CoDa thus offers several methods to estimate and correct for publication bias implemented in *metafor* (Carter et al., 2019; Viechtbauer, 2010). Publication bias can be detected in a funnel plot, which plots effect sizes against their standard errors (Duval & Tweedie, 2000). An asymmetry in the funnel plot indicates missing studies. CoDa implements three tests for funnel-plot asymmetry: the rank correlation test, Egger’s regression test, and the trim-and-fill method. We also provide adjustments for publication bias using the trim-and-fill method and the Henmi-Copas method (Begg & Mazumdar, 1994; Egger et al., 1997; Henmi & Copas, 2010).

#### Power analysis

One major benefit of meta-analysis is that the estimate of a population-level effect size can be used in statistical power analysis to calculate the sample size required in the next study that investigates the effect. An additional function of CoDa is that the effect-size estimate is used as input in a statistical power analysis to estimate the adequate sample size required in future studies to detect this effect size (see Fig. S4 in the Supplemental Material). The user can further customize these analyses by adjusting assumptions about the statistical test and the levels of α and β of these analyses. The power analyses are based on the R package *pwr* (Champely, 2020). This function of CoDa can help establish standards in the field for required sample sizes in future studies.

#### Additional visualizations

The CoDa platform also offers users the ability to explore the literature with two different visualization tools. The Citation Explorer facet displays the citation network of studies annotated in CoDa, which enables users to identify communities and topics within the history of research on human cooperation. The citation network models the articles included in the databank (for which a DOI could be found) and established links between the articles on the basis of their references. The citations were obtained from CrossRef and Microsoft Academics using the papers’ DOIs as entry points. Then, communities were identified according to their modularity (i.e., the density of the links within and between communities, using the Louvain community detection algorithm; Blondel et al., 2008). Results were then imported into the CoDa platform using R’s *visNetwork* library (Almende et al., 2019). The visualizations can be customized by users to explore studies using the information annotated in CoDa.

The platform also offers an Ontology Explorer (Fig. 4), which allows users to visualize and dynamically interact with the domain-specific schema of human-cooperation studies. Similar visualization approaches have been widely adopted in existing projects, such as metaBUS, and provide an overview of the relative frequency of investigation of specific topics (Bosco et al., 2020). In CoDa, all classes are subclasses of the generic class “Independent Variable,” which corresponds to the outer circle. The size of a circle represents the number of treatments annotated with that variable included in the databank. The user can hover on a circle to display the definition of a desired class (e.g., Personality) and additionally zoom in and obtain the labels and definitions of its subclasses. For example, in Figure 4, the observer can quickly see that punishment has been studied more frequently than personality and can zoom in to learn about the many different personality constructs that have been studied in relation to cooperation.

## Scientific Benefits of CoDa

CoDa offers many of the same outstanding benefits of individual meta-analyses, including estimating effect sizes, analyzing variation in effect sizes across a literature, and providing input for statistical power analyses (for review, see Gurevitch et al., 2018). Yet CoDa provides unique benefits beyond the traditional use of meta-analysis, including setting standards for sample sizes in an entire field, enhancing replication and reproducibility of meta-analyses, publication of null results, facilitating equivalent comparisons across studies, and a tool for exploring patterns across the results of several meta-analyses of research on cooperation.

The social sciences have always had a persistent problem of low statistical power and therefore high rates of Type II errors (i.e., the failure to reject false null hypotheses; Cohen, 1962, 1992). CoDa can directly contribute to reducing the number of Type II errors in the study of human cooperation. Statistical power analyses can be used to estimate desirable sample sizes to reduce Type II errors, but researchers are often unaware of the estimated population effect size to input into a priori power analyses. CoDa can be used to monitor the estimated effect sizes for all possible effect sizes in the literature on cooperation, which can set standards for sample sizes.

Specific research questions can suffer from publication bias (Ferguson & Heene, 2012). The “gold standard” for estimating publication bias is the comparison of study results from both published and unpublished studies. CoDa already contains unpublished effect sizes that were reported in published meta-analyses, and these can be used to make comparisons between published and unpublished study results. The platform also provides users the ability to estimate and correct for publication bias (Box 3b). Finally, researchers can submit their own unpublished (null) results to be included in CoDa.

Meta-analyses are time-consuming, involve many decisions (often unreported), and are therefore difficult to reproduce (Gurevitch et al., 2018). The CoDa platform allows researchers to document their data and analytic approach to a meta-analysis, which can be reported and subsequently reproduced by other users. Researchers can use CoDa to discover studies that are most comparable with their own research. For example, a researcher may want to find a study that has all male participants playing a public-goods game with a specific group size. Such a detailed search is possible with machine-readable publications, and comparing study results with a greater level of specificity can assist researchers in reporting how their study compares with existing research. Reviewers of manuscripts can also use CoDa to efficiently and quickly examine how a study compares with existing research.

## Limitations and Future of CoDa

There are some limitations of CoDa. To date, CoDa contains studies from 78 countries/regions (see the Supplemental Material), most of which were conducted in the United States (41%) or other societies that are Western, educated, industrialized, rich, and democratic (WEIRD; Henrich et al., 2010). To address this issue, we have included studies published in both Japanese and Chinese to expand the databank beyond Western societies. We are in the process of developing a mechanism for researchers around the world to submit their published or unpublished studies to CoDa to make them machine-readable and available on the platform. The submitted studies will be added to the databank after review by a team of editors.

CoDa does not provide an overall indicator of confidence in the body of evidence. Rather, decisions about confidence can be jointly informed by a variety of indicators provided in the meta-analytic output that estimate relevant dimensions of confidence, specifically, imprecision (i.e., 95% confidence interval of the meta-analytic estimate), inconsistency (i.e., heterogeneity indicators), and publication bias. However, CoDa does not provide an estimate of the risk of bias that specific study-design features might introduce in the meta-analysis (Appelbaum et al., 2018). Because the existing methods to assess risk of bias were built for other fields of research (e.g., involving clinical interventions; Higgins et al., 2011), a future direction for CoDa could be to develop standards to assess risk of bias within studies on cooperation. In the meantime, risk of bias can be minimized through several current and forthcoming features enabled in the CoDa research platform, such as including unpublished studies, allowing users to make decisions about inclusion/exclusion of specific study results, and enabling users to build moderator models, export the data, and perform analyses using different model specifications (Burgard et al., 2021).

Currently, tasks to develop CoDa, such as the search for studies, screening articles, and data extraction, have been conducted by trained domain experts. This requires a substantial time investment, which is a challenge to CoDa’s long-term sustainability. Technologies based on machine learning and natural language processing techniques have potential to (partly or fully) automatize such tasks (Marshall & Wallace, 2019). Existing projects have made use of such techniques, even if at initial stages, and show promise in terms of expediting research synthesis (e.g., Human Behaviour Change Project, Ganguly et al., 2018; Michie et al., 2017; NeuroSynth, Yarkoni et al., 2011). To date, however, the performance of these automated tools is suboptimal in terms of achieving the accuracy that is required for systematic reviews (Bosco et al., 2020; Jonnalagadda et al., 2015; Marshall & Wallace, 2019). The large corpus of manually curated studies in CoDa could prove useful to develop automated techniques to perform these tasks and to evaluate the accuracy of these approaches (Bosco et al., 2020).

At present, CoDa documents only observations about cooperation. We can expand the ontology and the data included in the databank to other topics and disciplines. More specifically, the ontology can be expanded to (a) capture more constructs and (b) define the relations between constructs. This could advance knowledge and theory. Indeed, adopting a shared conceptualization of constructs and their relationships, as done while generating ontologies, can serve as a basis not only for summarizing the current state of knowledge but also encouraging the development of new knowledge by integrating, developing, disambiguating, and resolving inconsistencies and evaluating theories (Gray, 2017; West et al., 2019; for an example of ontology-based theory representation in the behavioral change field, see Hale et al., 2020).

## Conclusion

We offer a resource for scientists and practitioners to search, explore, and compare empirical studies on human cooperation using social dilemmas. CoDa offers several functions that address the needs of scientists working in a rapidly expanding literature and for practitioners searching for evidence-based techniques to enhance cooperation. We developed an Ontology of Human Cooperation Studies, which can be used to represent the relations between study results, and we had experts annotate the literature and translate results contained in PDFs into a standardized machine-readable format. We applied state-of-the-art methods to build a search tool using an ontology that allows users to select and curate a set of studies to be used for on-demand meta-analysis, metaregression, publication bias assessment, and power analyses.

Machines can be built to better assist scientists in monitoring trends in the literature and to facilitate the processing and comparison of study results. Standardized methods of reporting study results hold the promise to usher forward a more automated solution to develop tools such as CoDa and on much larger scale. Machine-readable and ontology-based representations of scientific findings can complement traditional publications in scientific journals and provide additional benefits, such as enhancing their findability and accessibility and facilitating their computational processing. Scientific journals could establish editorial policies that would integrate this as part of the publication process. Machine-readable science can even lead to novel publishing formats, such as living meta-analytic reviews that update automatically when new data become available. Currently, living reviews are implemented in the medical sciences through groups of authors taking care of performing a baseline systematic search of the literature and committing to provide constant updates (e.g., every 6 months) to the resulting review (Elliott et al., 2017). Relying on a large annotated body of machine-readable linked data can represent a first step to make such an endeavor more sustainable. At the moment, CoDa offers a vision of how publishing research results in a standardized machine-readable format can lead to establishing institutions and tools that improve scientific practices and stimulate scientific progress.

## Supplemental Material

sj-pdf-1-pps-10.1177_17456916211053319 – Supplemental material for The Cooperation Databank: Machine-Readable Science Accelerates Research Synthesis Click here for additional data file. Supplemental material, sj-pdf-1-pps-10.1177_17456916211053319 for The Cooperation Databank: Machine-Readable Science Accelerates Research Synthesis by Giuliana Spadaro, Ilaria Tiddi, Simon Columbus, Shuxian Jin, Annette ten Teije, CoDa Team and Daniel Balliet in Perspectives on Psychological Science

## Transparency

*Action Editor:* Laura A. King

*Editor:* Laura A. King

*Team Members*

Sakura Arai, University of California, Santa Barbara

Roberta Ciulla, Palermo, Italy

Pascalle de Vos-Tamis, Radboud University

Yukako Inoue, Kochi University of Technology

Yasuyuki Kudo, Hitachi, Ltd. Research & Development  Group

Eleanor Lieberman, Harvard University

Catherine Molho, University of Amsterdam

Annika S. Nieper, Vrije Universiteit Amsterdam

Kateland Pahor, University of Queensland

Maria Isabela Rinderu, Vrije Universiteit Amsterdam

Claudia Smakaj, University of Bologna

Adam W. Stivers, Gonzaga University

Isabel Thielmann, University of Koblenz-Landau

Alisha Uebergang, University of Queensland

Olmo van den Akker, Tilburg University & University of  Amsterdam

Junhui Wu, Institute of Psychology, Chinese Academy of  Sciences

Mingliang Yuan, Beijing Normal University

## Notes

1. Software version numbers are not given throughout because we plan to perform regular updates, which will include updates to the current versions of all underlying software.

2. In a knowledge graph, all concepts (classes, relationships, and instances) have a *unique resource identifier* (URI; i.e., an HTTP identifier that can be accessed via the web). Namespaces can be used to shorten URIs; we use cdo: to represent terms in the ontology (e.g., cdo:Paper is < https://data.cooperationdatabank.org/vocab/class/Paper >), whereas we can use the id: namespace to represent instances of a class (e.g., id:ENG00840, which stands for https://data.cooperationdatabank.org/id/ENG00840 and represents an instance of the class cdo:Paper).
