# Supplementary material for: The Cooperation Databank: Machine-Readable Science Accelerates Research Synthesis
Source: Perspect Psychol Sci. 2022 May 17;17(5):1472–89. doi: 10.1177/17456916211053319 (PMC9442633; doi:10.1177/17456916211053319)
Supplement: sj-pdf-1-pps-10.1177_17456916211053319 – Supplemental material for The Cooperation Databank: Machine-Readable Science Accelerates Research Synthesis [file sj-pdf-1-pps-10.1177_17456916211053319.pdf]

Supplemental Material

“The Cooperation Databank:

Machine-Readable Science Accelerates Research Synthesis”

Giuliana Spadaro, Ilaria Tiddi, Simon Columbus, Shuxian Jin,

Annette C. M. ten Teije, CoDa Team, Daniel Balliet

Table of Contents

1. Search for Studies and Study Inclusion Criteria **2**

1.2 Study Inclusion Criteria 3

2. Annotation of Studies **7**

2.1 Training of Contributing Members 7

2.2 Annotation and Data Structure 8

2.3 Inter-rater Agreement: Annotation and Analyses 9

3. Effect Size Calculations **15**

4. The Dataset **19**

5. Analyses **28**

7. The CoDa Research Application and Platform **31**

8. References **34**

1. Search for Studies and Study Inclusion Criteria

1.1 Search for studies to be included in CoDa

Three English-speaking domain experts conducted systematic searches for English written documents in 2015 and 2018 using PsychInfo, Web of Science, and Google Scholar. Chinese documents were searched in November and December 2017 using CNKI, Wangfang Data, and CQVIP by one Chinese domain expert. The search for Japanese documents was conducted from July to December 2018 using Cinii by two Japanese domain experts. All domain experts held at least a master's degree and attended courses or performed research on cooperation.

The search log included the following key terms: Public goods dilemma*, Public good*, Public good* game*, Prisoner's dilemma*, Voluntar* contribut* experiment*, Voluntary contribution mechanism, Social dilemma, Mixed-motive game, Mixed-motive game*, Cooperation game (up to 2015), Resource dilemma*, Conditional cooperation (up to 2015), Interpersonal bargaining AND Experimental games (up to 2015), Matrix games, Cooperation AND Experiment. For documents published between 2016 and 2017, we additionally used the following terms: Common pool game, Give-some dilemma, Take-some dilemma, Give-some game, Take-some game. Equivalent terms were used for Japanese and Chinese searches. In order to limit the search output to only studies involving human samples, some searching strategies included the following specification: NOT Publication Name: "Theoretical Biology" NOT Publication Name: "Europhysics Letters" NOT Publication Name: "Physical Review" NOT Publication Name: "Computational". The keywords used for the search have been identified by domain experts. For English documents, which represent the majority of available documents, the records retrieved through each search log ranged from 4 to 813 (*Mdn* = 155).

Additionally, we performed a backward search of the social dilemma literature by reviewing articles cited by published reviews (Chaudhuri, 2011; Dawes, 1980; Pruitt & Kimmel, 1977; Wrightsman et al., 1972), meta-analyses (Balliet, 2010; Balliet et al., 2009, 2011, 2014; Balliet & van Lange, 2013; Balliet & Van Lange, 2013; Fiala & Suetens, 2017; Mengel, 2018; Pletzer et al., 2018; Rand, 2016; Sally, 1995), and books (Biel et al., 2008; Foddy, 2013; Henrich et al., 2005; Komorita, Parks, 1995; Liebrand, Messick, 1996; Rapoport et al., 1965; Suleiman et al., 2004). In addition, some documents were retrieved as they were cited in papers found using the above-mentioned search strategies. Finally, we sent announcements requesting published data to the Economic Science Association (ESA), European Association of Social Psychology (EASP), Judgement and Decision Making (JDM), European Association for Decision Making (EADM) listservs and on Twitter. Although this search strategy combined many different sources to enhance the comprehensiveness of the search output, such strategy was not peer-reviewed or cross-validated. Given the breadth of the systematic search, the domain experts did not perform two (or more) independent searches. Rather, they performed different search queries to improve time efficiency.

1.2 Study Inclusion Criteria

Screening the papers' title and abstract to identify potentially relevant records was carried out by the same domain experts who performed the systematic search. Final decisions about eligibility for inclusion in CoDa are done at the study level, based on the analysis of the full text of the paper, in original publication language. This means that for documents that report multiple studies (e.g., multi-study papers), only the eligible studies are included in the databank. Several standards were used to determine whether a study was eligible for inclusion (see Table S1 for an

overview of the criteria summarized according to guidelines provided by Atkinson and colleagues (2015).

(a) *Human participants*. The study was required to have a sample of human participants, with no further specific requirements (e.g., no age limitations). Thus, studies exclusively employing agent-based models or studies with other animals were excluded from CoDa.

(b) *Game type*. Cooperative behavior was assessed in a prisoner's dilemma, public goods game, or resource dilemma. Studies that involved variations of these game paradigms (e.g., asymmetrical and non-linear payoff functions, intergroup settings; Bornstein, 2003) were also eligible.

(c) *Reported cooperation*. The study reported the overall average amount of cooperative behavior or provided enough other quantitative information to calculate effect sizes in this sample. This could be calculated across all trials of the game or only for specific trials of the game.

Domain experts were instructed to annotate studies that met the above-mentioned standards. Decisions about eligible studies were not made by two (or more) independent domain experts. Rather, the training protocol encouraged the experts to discuss any uncetainty about study inclusion decisions with the core CoDa team. During the annotation of studies, it was discovered that, although some studies were identified as eligible, they did not report the overall average cooperation or enough information to compute effect sizes. Even though these studies cannot be included in meta-analyses, these studies are included in the platform. This is because these studies can still be reported to users as having measured cooperation or other specific variables of interest, and this can be included as output during a search for studies. Contrary to

what is recommended in the PRISMA statement (Page et al., 2021), at the time of the screening, we did not keep track of which studies met many but not all inclusion criteria (i.e., near misses).

In our search, we excluded studies that employed methods that were not highly similar to social dilemma paradigms. This included many other economic games, such as the Trust Game, Ultimatum Bargaining Game, or Dictator Game (for an overview see Thielmann et al., 2020). For example, we did not include the trust game, because this game has an important distinction from the vast majority of prisoner dilemmas (i.e., the trust game involves sequential decision making, while the Prisoner's Dilemma involves simultaneous decision making). Of course, the trust game is highly relevant to the study of human cooperation, however, a search criterion restricted to social dilemmas already output thousands of studies to annotate and we needed to demarcate a starting point for CoDa. Importantly, the databank can be expanded to accommodate all the situations and paradigms researchers use to study cooperation (and other social behaviors too).

Table S1

Criteria for Initial Screening and Final Inclusion of Records

Steps	Criteria
**Initial screening for relevance**	
Elements of reports used in initial screening decisions	Title and abstract
Criteria for passing from first to second screen	Empirical study using a social dilemma paradigm (not theoretical reviews or agent-based simulations)
**Final inclusion criteria**	
Researched variables	Cooperative behavior
Participants	Human participants
Researched settings	Lab, field, online, lab-in-the-field, classroom
Dates	Published or available online up to 2017[1]
Publication status	Published as Journal Article or available as Working Paper, Dissertation, Thesis
Treatment of studies reported in foreign languages	Full text assessment and annotation of Chinese and Japanese records performed by native speakers. Exclusion of records in different languages
Adequate reporting	Average amount of cooperative behavior, effect size in relation to cooperation, or statistical information that allows to compute effect size

[1] For a number of reasons, some studies conducted after the inclusion date (n = 28) were annotated and are currently included in the dataset.

2. Annotation of Studies

2.1 Training of Contributing Members

The annotation of studies was performed by domain experts, who proved to have familiarity with experimental studies using social dilemmas paradigms and meta-analytic techniques. All contributing members underwent a standard training procedure before annotating studies. In general, this procedure involved one week of full-time work annotating studies under the supervision of Isabela Maria Rinderu (2015-2017) and Giuliana Spadaro (2017-2020). The training procedure consisted of four steps:

(1) Instruction about the Codebook and annotation scheme;

(2) Annotating sample and study characteristics from a set of previously annotated studies;

(3) Annotating quantitative information from the same set of previously annotated studies;

(4) Complete annotation of studies from a set of previously annotated studies;

Each of these steps was followed by a meeting with the supervisor, to solve all discrepancies and answer questions. Usually, the standard training occurred face-to-face, but a few remote training sessions were also performed. After the training session, the trainee continued to annotate already annotated studies until the number of discrepancies was significantly reduced and they reported to feel confident with the task. During the entire duration of their annotation work, they had regular meetings with the supervisor to discuss and solve any eventual remaining questions. For two contributing members whom provided an extensive annotation effort and full-time commitment for over one year of work (i.e., Shuxian Jin and Mingliang Yuan), the training procedure involved additional iterations to maximize the accuracy and confidence of the annotation work.

2.2 Annotation and Data Structure

As a general rule, we annotated overall mean values, standard deviations, and effect sizes as reported in the document. However, when the information was not directly reported, we made several decisions to obtain comparable statistical information to calculate effect sizes.

First, all means and standard deviations of cooperation were computed by pooling multiple data points as

$$Overall\ M_{(COOP)} = \frac{\sum n_i M_i}{\sum n_i},$$

$$SD_{pooled} = \sqrt{\frac{(n_1 - 1)S_1^2 + (n_2 - 1)S_2^2 + \ldots + (n_k - 1)S_k^2}{n_1 + n_2 + \ldots + n_k - k}}.$$

To calculate an overall observed cooperation in a study using reports of cooperation across multiple treatments, we assumed equal sample size (*n*) across treatments when the sample size per cell was not reported.

Second, when regressions were performed, we decided to only include estimates from simple linear regression with a single predictor (Borenstein et al., 2009; Lipsey & Wilson, 2000). Thus, estimates obtained from multiple, hierarchical, and multi-level regression analyses were not annotated or used to calculate effect sizes.

After completing the annotation of a study, the studies were further divided into sub-studies and annotated when at least one of the following criteria was met: (a) the study was replicated in multiple countries (e.g., from cross-cultural studies, Herrmann et al., 2008), (b) the study reported observations in multiple games (e.g., Parks, 2000), (c) the study was replicated in multiple labs (e.g., from multi-lab replication projects, Bouwmeester et al., 2017), and (d) the study had asymmetric endowment size (e.g., studies manipulating inequality in wealth, Van Dijk & Wilke, 1994). In this case, one original study would result in one plus *n* additional annotated

records (i.e., sub-studies). For example, a cross-cultural study comparing cooperative behavior of participants from two different countries (e.g., United States and Japan) would result in three annotated records (one original study plus two sub-studies). In this instance, in addition to the original study (with annotated sample size N_{overall} and M_{overall} and SD_{overall} of cooperation) two more sub-studies would be added (with annotated sample size N_{USA} and M_{USA} and SD_{USA} of cooperation in the study conducted in the United States, and with sample size N_{JPN} and M_{JPN} and SD_{JPN} of cooperation in the study conducted in Japan). The annotation of multiple sub-studies was performed to provide more fine-grained data to be used for meta-analyses. Thus, the approach to annotate studies, represent studies in the data model, and meta-analyze studies are all applicable to sub-studies. In fact, when we refer to “studies” in the manuscript, this also includes sub-studies.

Overall, the data provided in CoDa are organized according to a hierarchical structure. As mentioned above, a paper could contain one or more eligible studies, and they can, in turn, be further divided into sub-studies. For each of those studies (or sub-studies) one or more variables can be manipulated or measured to be examined in relation to cooperation, resulting in one or more effect sizes per study. The hierarchical structure of the data can be accounted for using multi-level meta-analytic techniques. When performing analyses on the research platform, overall study and sub-studies are never both included in an analysis, and the overall study is automatically excluded if sub-studies are included in the selection.

2.3 Inter-rater Agreement: Annotation and Analyses

To estimate the inter-coder agreement, we selected 10% of the documents to be re-annotated by a second person. At the time, more than 1,600 English, Chinese, and Japanese documents were annotated by 15 contributing members. In order to include a representative

sample of documents annotated by each contributing member, we selected documents using a stratified method. This approach was used to randomly select 10% of documents annotated by each contributing member. This selected sample of studies was then re-annotated to estimate inter-rater agreement. In total, 160 documents were chosen and re-annotated by five members from our team, including two native Chinese speakers and two native Japanese speakers. Reliability was assessed by comparing the re-annotations with those of the original annotators.

We re-annotated all 44 study characteristics (i.e., 21 continuous variables, 23 categorical variables). We estimated inter-rater agreement using Krippendorff's α (Hayes & Krippendorff, 2007; Krippendorff, 2011). The general formula to calculate α is

$$\alpha = 1 - \frac{D_O}{D_e},$$

where D_o is a measure of the observed disagreement and D_e is the disagreement which can be expected as a result of chance. When there is perfect agreement between annotators, observed disagreement $D_o = 0$ and $\alpha = 1$ (i.e., perfect reliability). However, when annotators agree only as a product of chance, observed disagreement $D_o = D_e$ and $\alpha = 0$ (i.e., perfect unreliability (Krippendorff, 2011). Krippendorff's α can estimate the degree of agreement for different measurements (e.g., nominal, ratio), and can assess reliability when there exist missing values (such as when specific information was not reported in the paper).

Additionally, for all categorical variables, we calculated the percentage of agreement as an additional estimate of inter-rater reliability. This index is calculated as the proportion of units with matching descriptions on which two observers agree. The main reason for including this information is that the underlying distribution of a variable can impact assessments of inter-rater agreement, and when distributions are seriously skewed (e.g., specific categories are highly common), then the percentage of agreement may reflect the actual level of inter-rater agreement

(Jones et al., 1983). In CoDa, some of the categorical variables were found to have little variation in annotation across studies (see column “Most frequent level (%)” in Table S2), which indicates that percentage of agreement is a more suitable method to estimate agreement.

The results show that most variables scored a high level of inter-rater agreement. However, the results of inter-rater agreement revealed a few variables with inadequate levels of agreement. As a result, we re-annotated two categorical variables using a lower number of levels, which substantially improved the estimates of inter-rater agreement (i.e., recruitment method, matching protocol). We also removed three variables that had unacceptably low inter-rater agreement (i.e., number of trials, number of blocks, feedback). This effort resulted in a set of 39 variables (17 continuous and 22 categorical) on with high inter-rater agreement (see Table S2).

Compared to the continuous variables, the categorical variables presented lower rates of agreement. This finding is not surprising because categorical variables are the most dependent on the annotation scheme (e.g., as compared to numeric values such as total sample size or mean age of the participants). However, categorical variables with low to medium levels of Krippendorff’s α (e.g., published, symmetry, and discussion) showed on the other hand a percentage of agreement higher than 90%, suggesting that the low α can be a result of little variation across levels of the variables. The variable “source of country” displayed both a poor Krippendorff’s α and percentage of agreement. We believe that this is due to the hierarchical and non-mutually exclusive structure of the possible levels of this variable that is not optimal to provide convergent annotations. Indeed, the level of inter-coder agreement of the related variable “country of data collection” were high ($\alpha = 0.92$, agreement = 93.7%), suggesting that there was no disagreement in how country was annotated among different annotators. We, thus, decided to not remove this variable from CoDa.

Table S2

Estimates of Inter-Coder Agreement

Concept	Values	Krippendorff's α	Most frequent level (%)	Agreement %
Study Meta-data				
Published	Published, Doctoral Dissertation, Working paper, Master's thesis, Raw data	0.66	Published (97.1%)	98.50%
Sample Characteristics				
Academic discipline	Other, Economics, Psychology, Mixed, Sociology	0.70	999 (49.5%)	79.10%
Country	[string]	0.92	USA (36.9%)	93.70%
Highest age	[int]	1.00	-	-
Lowest age	[int]	1.00	-	-
Mean age	[double]	1.00	-	-
Proportion of males	[double]	0.92	-	-
Recruitment method	Participant pool, MTurk, Other	0.76	Participant pool (49.6%)	85.50%
Source of Country	Specified country, Most authors, All authors, Multiple countries	0.65	Specified country (84.1%)	90.30%
Source of year of data collection	Received/Submitted, Conducted, Published, Accepted, Presented, Working paper published, Available online	0.78	Received/Submitted (55.8%)	85.90%
Student sample	[bool]	0.89	TRUE (85.3%)	97.30%
Total N	[int]	0.93	-	-
Year of data collection	[int]	0.98	-	-

Concept	Values	Krippendorff's α	Most frequent level (%)	Agreement %
Study Characteristics				
Continuous vs step-level public goods	Continuous, Step-level	0.78	N/A (53.4%)	87.90%
Deception	[bool]	0.74	FALSE (71.2%)	89.10%
Discussion	Bi-directional, Uni-directional, Absent	0.68	Absent (93.2%)	96.60%
Experimental setting	Lab, Class, Lab in the field, Field, Natural experiment, Other	0.75	Lab (83.5%)	93.50%
Game incentive	Hypothetical, Monetary, Non-monetary, Monetary lottery, Non-monetary lottery	0.77	Monetary (76.1%)	92.10%
Game type	Prisoner's Dilemma Game, Public Goods Game, Resource Dilemma, Other	0.75	Public Goods Game (43.7%)	82.90%
Group size	[int]	0.97	-	-
Highest choice option	[int]	0.92	-	-
K index	[double]	0.91	-	-
Known endgame	[bool]	0.78	TRUE (73.4%)	90.70%
Lowest choice option	[int]	0.71	-	-
Matching	Stranger, Partner	0.84	Stranger (49.8%)	91.60%
MPCR	[double]	0.95	-	-
Number of choices	[int]	0.96	-	-
One-shot vs repeated	Repeated, One-shot	0.81	FALSE (52.2%)	90.20%
Real partner	Real, Hypothetical, Deception	0.77	Real (70.2%)	89.20%
Repeated one-shot game	[bool]	0.69	N/A (50.5%)	80.50%

	Concept	Values	Krippendorff's α	Most frequent level (%)	Agreement %
	Sequentiality	Simultaneous, Sequential turn-taking	0.66	Simultaneous (89.3%)	93.60%
	Show-up fee	Paid, Course credit, Non-monetary, Absent	0.73	Paid (63.2%)	84.90%
	Symmetry	[bool]	0.59	TRUE (93.1%)	94.60%
	Threshold	[double]	0.82	-	-
Quantitative Study Results					
	Overall mean of contributions	[double]	0.97	-	-
	Overall proportion of cooperation	[double]	0.95	-	-
	Overall standard deviation of contributions or withdrawals	[double]	0.94	-	-
	Percentage of endowment contributed	[double]	0.79	-	-
	Trial of cooperation	First trial, Last trial, First and last trials, Other trials, All trials	0.69	All trials (92.3%)	94.90%

Note. For categorical variables, both Krippendorff's α and percentage of agreement are reported. [bool] = values for this variable are Boolean; [double] = Admitted values for this variable are numeric values with decimals allowed; [int] = Admitted values for this variable are numeric integer variables; N/A = Not applicable variable; 999 = missing information (not reported in the paper).

3. Effect Size Calculations

Standardized effect sizes express the relationship between two variables on a standardized metric. We computed effect sizes for each cdo:Observation for which sufficient information was available. The effect size can express the relationship between a single continuous variable and the measure of cooperation (if cdo:Observation has a single cdo:Treatment) or the difference in cooperation between two cdo:Treatment in a cdo:Observation. In the dataset, the direction of effect sizes comparing two cdo:Treatment corresponds to the order of the treatments. However, effect sizes are aligned with the user-defined selection in the research platform.

Standardized effect sizes were obtained directly from papers or computed from a range of statistics annotated from the papers. We computed effect size estimates in the form of Cohen's *d* and the correlation coefficient *r*, as well as their accompanying variances. Most effect size computations were conducted using the R package *esc* (Lüdecke, 2019), which implements formulas described in Lipsey and Wilson (2000).

Cohen's *d*. Cohen's *d* is a standardized measure of the difference between two groups on a continuous outcome variable (Cohen, 1988). We computed Cohen's *d* for between-group comparisons using multiple formulas. Whenever an estimate could be computed from multiple combinations of inputs, we applied a hierarchy of algorithms to select the most reliable computation (Lipsey & Wilson, 2000). Where means $\bar{X}_i$, standard deviations s_i, and sample sizes n_i were available for each treatment, *d* was computed as

$$d = \frac{\bar{X}_1 - \bar{X}_2}{s_{pooled}},$$

where s_{pooled} is the pooled standard deviation,

$$s_{pooled} = \sqrt{\frac{(n_1 - 1)s_1^2 + (n_2 - 1)s_2^2}{n_1 + n_2 - 2}}.$$

Alternatively, Cohen's *d* can also be estimated from the *t* statistic computed by comparing two group means. In this case, *d* is given by

$$d = t\sqrt{\frac{n_1 + n_2}{n_1 n_2}}.$$

However, sometimes only the overall sample size *N* across both groups or, more likely, the degrees of freedom of the *t* test may be available, where $N = df + 2$. In this case, *d* can be approximated as

$$d = 2 \times \frac{t}{\sqrt{N}}.$$

The variance of the Cohen's d, s_d^2 is used in weighting the studies in the meta-analysis. For between-group designs, it is computed from the sample sizes of each group and the effect size estimate. When only *N* is available, equal-sized groups are assumed, such that $n_1 = n_2 = N/2$. Then,

$$s_d^2 = \frac{n_1 + n_2}{n_1 n_2} + \frac{d^2}{2 \times (n_1 + n_2)}.$$

A second case occurs when the outcome is dichotomous. In this case, Cohen's *d* can be obtained by computing the odds ratio *OR* from proportions p_i and sample sizes n_i, then converting the odds ratio to *d*,

$$OR = \frac{p_1 n_1 \times (1 - p_2) n_2}{p_2 n_2 \times (1 - p_1) n_1},$$

$$d = \frac{log\ OR}{\pi/\sqrt{3}},$$

$$s_d^2 = \frac{p_1 n_1 + (1 - p_2) n_2 + p_2 n_2 + (1 - p_1) n_1}{\pi^2/3}.$$

For within-subjects comparisons, we only implement effect size estimates for continuous outcomes. We are not aware of a method to estimate Cohen's *d* for within-subjects comparisons of proportions. For comparisons of means, several approaches of been proposed (Westfall, 2016). All approaches require the correlation between measures *r*, which is rarely reported and which we therefore did not annotate. To compute *d*, we use change score standardization, which uses the standard deviation of the change score, and impute *r* = 0.5 (*Variance of Cohen's d for within Subjects Designs*, 2017). This function was implemented in R because the standard deviation computed by *esc* uses slightly different formula.

$$d = \frac{\bar{X}_1 - \bar{X}_2}{s_{change}},$$

$$s_{change} = \sqrt{s_1^2 + s_2^2 - 2 \times r \times s_1 s_2},$$

$$s_d^2 = \frac{1}{n} + \frac{d^2}{2 \times n}.$$

Correlation coefficient *r*. The correlation coefficient *r* is a measure of the association between two continuous variables. It is often reported in papers, and whenever possible, we used the reported value of *r* and computed the variance as

$$s_r^2 = \frac{1 - r^2}{n - 2}.$$

When possible, we also provide estimates of *r* for data with categorical predictors. Specifically, where Cohen's *d* for between-group comparisons could be computed from means or *t* statistics, we convert from *d* to *r*,

$$r = \frac{d}{\sqrt{d^2 + 1/\big(p \times (1-p)\big)}},$$

$$s_r^2 = \frac{s_d^2}{s_d^2 + 1/(p \times (1-p))},$$

where p is the proportion of participants in the first treatment,

$$p = \frac{n_1}{n_1 + n_2}.$$

We do not provide conversions from d to r for within-subjects designs and data reported as proportions, as no reliable formulas exist. Similarly, we do not provide conversions from r to d.

4. The Dataset

Table S3

Number of Instances for Each Class in CoDa and Links to Existing Datasets

Class	Definition	Number of instances	Linked datasets	Number of links
cdo:Paper	A document reporting the methods and results of one or more studies.	1,809	-	-
cdo:Study	A sample of observations of a phenomenon in either a controlled (e.g., random assignment) or natural setting.	2,636	-	-
cdo:DOI	Digital Object Identifier that is a string of numbers, letters, and symbols that can be used to identify a document and link to it on the web.	1,327	-	-
foaf:Person	Authors identified by name and surname.	3,754	ORCID	66
cdo:IndependentVariable	A variable that is measured or manipulated and then related to a dependent variable, such as cooperation.	230	MeSH	103
cdo:Treatment	Treatments define when observations of a phenomenon occur in different contexts, such as multiple levels of a manipulated independent variable (IV).	14,141	-	-
cdo:Observation	Effects (i.e., either an association between a continuous IV and an outcome variable, or a contrast between two levels of a categorical IV on an outcome variable).	13,934	-	-
cdo:CountryModerator	Country-level variables retrieved from open access databases (e.g., World Values Survey, World Bank), to be used as covariates in meta-regressions.	97	-	-
cdo:Country/Region	Country or region where the data collection took place.	78	Wikidata	75

Table S4

Descriptive Information about the Annotated Independent Variables

Independent Variable	n	Boolean variables	Numerical variables		Categorical variables (most frequent values)		
		TRUE %	*Mdn*	Range	First	Second	Third
Academic discipline	18	–	–	–	Others (11)	Economics (5)	Psychology (2)
Academic grade	3	–	6	[4, 8]	–	–	–
Academic grade level	7	–	–	–	Junior (3)	Senior (3)	Middle (1)
Academic performance	2	–	–	–	–	–	–
Age	48	–	–	–	–	–	–
Age cohort	24	–	–	–	Old (11)	Young (11)	Middle (2)
Anchor	13	–	–	–	High cooperation (5)	Low cooperation (5)	Absent (3)
Anonymity	1	–	–	–	–	–	–
Anonymity manipulation	207	–	–	–	Low (99)	High (95)	Medium (13)
Assigned endowment	36	58%	–	–	–	–	–
Behavior in different game	23	–	–	–	Dictator Game (11)	Prisoner's Dilemma Game (3)	Other Game (2)
Behavior in different other game	3	–	–	–	BT game (2)	Risk-taking game (1)	–
Block of cooperation	362	–	2	[1, 10]	–	–	–
Block of cooperation (ordinal)	87	–	–	–	First (32)	Last (32)	Other (23)
Cognitive load treatment	20	50%	–	–	–	–	–
Communication content	204	–	–	–	Promise (97)	Other (43)	Irrelevant (27)
Communication occurrence	377	–	–	–	Ongoing (234)	One-shot (143)	–
Communication treatment	633	61%	–	–	–	–	–
Communication type	380	–	–	–	Written (219)	Verbal (151)	Nonverbal (10)
Comprehension of the game	1	–	–	–	–	–	–
Comprehension of the game level	2	–	–	–	High (1)	Low (1)	–
Conflict index	427	–	–	–	K index (311)	Other (58)	b/c ratio (23)
Conflict index value	435	–	0.45	[-13, 58]	–	–	–
Conflict level	398	–	–	–	High (144)	Low (144)	Medium (110)
Context frame	210	–	–	–	Cooperative (89)	Competitive (63)	Generic (50)
Continuation probability	36	–	0.83	[0, 1]	–	–	–
Continuation probability level	26	–	–	–	High (11)	Low (11)	Medium (4)
Costly monitoring	14	71%	–	–	–	–	–

Independent Variable	n	Boolean variables	Numerical variables		Categorical variables (most frequent values)		
		TRUE %	*Mdn*	Range	First	Second	Third
Criticality level	22	–	–	–	Low (11)	High (10)	Medium (1)
Decision maker	342	–	–	–	Individual (290)	Group (52)	–
Decision time	6	–	–	–	Fast (3)	Slow (3)	–
Decision time (correlation)	1	–	–	–	–	–	–
Degree of friendship	2	–	–	–	–	–	–
Descriptive norm	16	–	–	–	High Cooperation (6)	Low Cooperation (6)	Medium Cooperation (3)
Emotion	111	–	–	–	Neutral (25)	Anger (19)	Guilt (11)
Emotion intensity	6	–	–	–	Low (4)	High (2)	–
Emotion manipulation	136	75%	–	–	–	–	–
Emotion valence	40	–	–	–	Positive (18)	Negative (16)	Neutral (6)
Endogenous leadership	147	–	–	–	Exogenous (110)	Endogenous (37)	–
Endogenous motivational orientation	13	–	–	–	Cooperative (6)	Individualistic (6)	Competitive (1)
Endogenous position	292	9%	–	–	–	–	–
Endogenous threshold	15	33%	–	–	–	–	–
Endowment level	230	–	–	–	High (83)	Low (82)	Medium (65)
Endowment size	581	–	40	[1, 70000]	–	–	–
Entitativity	4	–	–	–	–	–	–
Entitativity level	2	–	–	–	High (1)	Low (1)	–
Ethnicity (US)	64	–	–	–	White (30)	Black or African American (21)	Other (9)
Exit option	11	55%	–	–	–	–	–
Expectations	177	–	–	–	–	–	–
Expectations level	56	–	–	–	High (27)	Low (27)	Medium (2)
Experimental setting	23	–	–	–	Lab (12)	Field (4)	Online (4)
External MPCR	4	–	5	[2, 12]	–	–	–
External MPCR level	4	–	–	–	Medium (2)	High (1)	Low (1)
Feedback content	289	–	–	–	Choice (214)	Earnings (31)	Failure (15)
Feedback target	362	–	–	–	Individual (151)	Absent (94)	Group (87)
Fixed resource	2	50%	–	–	–	–	–
Focal participant has punished	18	89%	–	–	–	–	–
Focal point frame	22	77%	–	–	–	–	–

Independent Variable	n	Boolean variables	Numerical variables		Categorical variables (most frequent values)		
		TRUE %	*Mdn*	Range	First	Second	Third
Gain-Loss frame	88	–	–	–	Gain (44)	Loss (44)	–
Game incentive	85	–	–	–	Monetary (54)	Hypothetical (26)	Non-monetary Material (5)
Game type	103	–	–	–	Other Game (38)	Prisoner's Dilemma Game (30)	Resource Dilemma (8)
Gender	677	–	–	–	Male (340)	Female (337)	–
Gender of the partner	50	–	–	–	Female (25)	Male (25)	–
Gender role	8	–	–	–	Career-oriented (4)	Tradition-oriented (4)	–
Give-Take frame	137	–	–	–	Give-some (74)	Take-some (63)	–
Gossip	12	–	–	–	Present (7)	Absent (5)	–
Group size	394	–	4	[1, 324]	–	–	–
Group size (correlation)	4	–	–	–	–	–	–
Group size level	323	–	–	–	Low (136)	High (131)	Medium (56)
Group type	414	–	–	–	Experimentally induced group (225)	Natural group (189)	–
Group variability	24	–	–	–	Low (13)	High (11)	–
Heterogeneous ethnicity	57	65%	–	–	–	–	–
Heterogeneous gender	639	52%	–	–	–	–	–
Highest choice option	15	–	6	[1, 500]	–	–	–
Highest choice option level	15	–	–	–	Low (7)	High (6)	Medium (2)
Hormone	5	–	–	–	Testosterone (4)	Cortisol (1)	–
Hormone level	3	–	–	–	High (1)	Low (1)	Medium (1)
Hormones administration	14	–	–	–	Oxytocin (6)	Placebo (6)	Vasopressin (2)
Identification	25	–	–	–	–	–	–
Identification level	22	–	–	–	High (11)	Low (11)	–
Income tax	7	71%	–	–	–	–	–
Income tax rate	7	–	0.5	[0, 1.3]	–	–	–
Income tax rate level	4	–	–	–	High (2)	Low (2)	–
Individual difference	2051	–	–	–	Social value orientation (556)	Trust propensity (200)	Openness to experience (91)
Individual difference level	164	–	–	–	High (78)	Low (78)	Medium (8)
Individual difference measure	1997	–	–	–	Triple Dominance Measure (Van Lange, 1997) (304)	Other (249)	HEXACO Personality Inventory-Revised (Lee & Ashton, 2006) (118)

Independent Variable	n	Boolean variables	Numerical variables		Categorical variables (most frequent values)		
		TRUE %	*Mdn*	Range	First	Second	Third
Individual MPCR	6	–	7.5	[2, 15]	–	–	–
Individual MPCR level	4	–	–	–	High (2)	Low (2)	–
Institution type	135	–	–	–	Taxation (51)	Punishment (42)	Other (20)
Institutional choice	186	–	–	–	Endogenous (122)	Exogenous (64)	–
Institutional choice mechanism	122	–	–	–	Vote (103)	Choice (10)	Migration (9)
Intentions	5	–	–	–	–	–	–
Intergroup competition	169	–	–	–	Individual group (73)	intergroup competition (40)	intergroup comparison (38)
Iterated pre-programmed cooperation rate	189	–	–	–	0.5 (51)	1 (38)	0 (29)
Iterated pre-programmed cooperation rate level	152	–	–	–	High (70)	Low (65)	Medium (17)
Iterated strategy	455	–	–	–	Preprogrammed cooperation rate (185)	Predominantly cooperative (64)	Tit-for-tat (62)
Knowledge of experimental games	14	50%	–	–	–	–	–
Knowledge of experimental games (correlation)	1	–	–	–	–	–	–
Knowledge of group membership	417	–	–	–	Common (369)	Unilateral (36)	Unknown (12)
Knowledge of partner's prior behavior	112	–	–	–	Absent (32)	Noncooperative (27)	Present (27)
Known endgame	74	58%	–	–	–	–	–
Leader's behavior	29	–	–	–	Cooperative (15)	Noncooperative (14)	–
Leader's characteristic	37	–	–	–	Strong (12)	Female (5)	Male (5)
Leadership	166	72%	–	–	–	–	–
Leadership assignment rule	143	–	–	–	Random Appointment (83)	Elected (23)	Authority (13)
Leadership role	44	–	–	–	Leader (25)	Follower (19)	–
Lottery incentive	83	–	–	–	–	–	–
Lottery punishment incentive	698	3%	–	–	–	–	–
Lottery reward incentive	120	4%	–	–	–	–	–
Lowest choice option	13	–	1	[0, 1]	–	–	–
Lowest choice option level	2	–	–	–	High (1)	Low (1)	–
Matching	109	–	–	–	Stranger (54)	Partner (51)	Partner's choice (4)
Minimum contribution	79	–	–	–	Mandatory Minimum (58)	Suggested Minimum (21)	–
Minimum contribution level	43	–	–	–	High (24)	Low (16)	Medium (3)

Independent Variable	n	Boolean variables	Numerical variables		Categorical variables (most frequent values)		
		TRUE %	*Mdn*	Range	First	Second	Third
Minimum contribution value	64	–	0.28	[0, 1.2]	–	–	–
Monitoring cost	10	–	–	–	High (4)	Medium (4)	Low (2)
Motivational orientation	57	–	–	–	Cooperative (23)	Competitive (21)	Individualistic (13)
MPCR	227	–	–	–	0.3 (43)	0.5 (36)	0.6 (24)
MPCR level	141	–	–	–	Low (59)	High (54)	Medium (28)
MRS	20	–	1.4	[0.1, 4.75]	–	–	–
MRS level	20	–	–	–	Medium (16)	High (2)	Low (2)
Nationality or region	80	–	–	–	USA (16)	CHN (9)	JPN (9)
Noise	37	59%	–	–	–	–	–
Normative beliefs	7	–	–	–	–	–	–
Number of blocks	84	–	1	[1, 15]	–	–	–
Number of blocks level	2	–	–	–	High (1)	Low (1)	–
Number of choices	15	–	6	[2, 501]	–	–	–
Number of choices level	15	–	–	–	Low (7)	High (6)	Medium (2)
Number of decision makers	322	–	4	[1, 324]	–	–	–
Number of trials	102	–	20	[1, 300]	–	–	–
Number of trials level	37	–	–	–	High (21)	Low (12)	Medium (4)
One-shot strategy	81	–	–	–	Prior contribution (79)	Prior withdrawal (2)	–
One-shot strategy value	82	–	0.49	[0, 1]	–	–	–
One-shot vs repeated	85	–	–	–	Repeated (46)	One-shot (39)	–
Optional communication	373	13%	–	–	–	–	–
Ostracism	56	57%	–	–	–	–	–
Other game type	39	–	–	–	Anticommons (6)	Prisoner's Dilemma (Fear-no greed) (4)	Prisoner's Dilemma (Greed-no fear) (4)
Other values	17	–	–	–	Authority (1)	Authority/Respect (1)	Care (1)
Participant's behavior level	23	–	–	–	High (11)	Low (11)	Medium (1)
Participant's own behavior (correlation)	2	–	–	–	–	–	–
Partitioned resource	8	50%	–	–	–	–	–
Partner choice	91	–	–	–	Exogenous (80)	Endogenous (8)	Migration (3)
Partner perception	138	–	–	–	Prosocial/Trustworthy (63)	Moral (17)	Attractive (15)

Independent Variable	n	Boolean variables	Numerical variables		Categorical variables (most frequent values)		
		TRUE %	*Mdn*	Range	First	Second	Third
Partner perception level	88	–	–	–	High (39)	Low (34)	Control (15)
Partner selection	24	–	–	–	Present (13)	Absent (11)	–
Partner type	78	–	–	–	Computer (38)	Stranger (29)	Peer (7)
Partner's behavior (correlation)	17	–	–	–	–	–	–
Partner's behavior level	2	–	–	–	High (1)	Low (1)	–
Partner's emotion	20	–	–	–	Neutral (7)	Anger (6)	Disappointment (3)
Partner's emotion display	25	–	–	–	Other (14)	Facial (11)	–
Partner's emotion manipulation	25	–	–	–	–	–	–
Partner's emotion valence	5	–	–	–	Negative (2)	Positive (2)	Neutral (1)
Partner's gender is known	501	63%	–	–	–	–	–
Partner's group membership	474	–	–	–	Ingroup (221)	Outgroup (126)	Stranger (116)
Perceived criticality	8	–	–	–	–	–	–
Physical proximity	6	–	–	–	High (3)	Low (3)	–
Political ideology	5	–	–	–	–	–	–
Position in game	202	–	2	[1, 8]	–	–	–
Power level	123	–	–	–	High (56)	Low (55)	Control (12)
Power manipulation method	90	–	–	–	Endowment (47)	Benefit (22)	Punishment (12)
Power type	122	–	–	–	Structural (112)	Experiential (8)	Conceptual (2)
Preference for conditional cooperation	51	–	–	–	Conditional cooperators (14)	Freeriders (14)	Others (13)
Primed construct	113	–	–	–	Other (30)	Neutral (29)	Cooperation (20)
Private account return	17	–	–	–	1 (5)	2 (3)	12 (2)
Private account return level	13	–	–	–	Low (7)	High (6)	–
Psychopathology	34	–	–	–	Control (13)	Depression (7)	Schizophrenia (4)
Public Good-Bad frame	22	–	–	–	Public bad (11)	Public good (11)	–
Punishment agent	725	–	–	–	Peer (429)	Institution (236)	Network (20)
Punishment distribution rule	685	–	–	–	Deductive (680)	Redistributive (5)	–
Punishment effectiveness	447	–	0.33	[0, 4]	–	–	–
Punishment incentive	710	–	–	–	Monetary (595)	Non-monetary social (47)	Hypothetical (42)
Punishment iterations	707	–	1	[1, 44]	–	–	–
Punishment probability	818	–	1	[0, 1]	–	–	–
Punishment rule	281	–	–	–	Contribution-based (183)	Rank-based (86)	Outcome-based (5)

Independent Variable	n	Boolean variables	Numerical variables		Categorical variables (most frequent values)		
		TRUE %	*Mdn*	Range	First	Second	Third
Punishment treatment	814	90%	–	–	–	–	–
Real communication	367	77%	–	–	–	–	–
Real partner	7	–	–	–	Real (4)	Hypothetical (3)	–
Rebate vs refund	42	–	–	–	Full refund (18)	Absent (15)	Partial rebate (9)
Relationship with the partner	82	–	–	–	Stranger (38)	Friend (33)	Acquaintance (10)
Religiosity	16	–	–	–	–	–	–
Religiosity operationalization	41	–	–	–	Religious affiliation (21)	Religious attendance (9)	Religious beliefs (9)
Religious exposure level	23	–	–	–	High (11)	Low (9)	Medium (3)
Replenishment rate	10	–	1.14	[1, 1.88]	–	–	–
Replenishment rate level	10	–	–	–	High (4)	Low (4)	Medium (2)
Resource size	10	–	180	[6, 1500]	–	–	–
Resource size level	27	–	–	–	High (10)	Low (10)	Medium (7)
Restart	2	–	–	–	First (1)	Last (1)	–
Restricted communication	375	50%	–	–	–	–	–
Reward agent	145	–	–	–	Peer (66)	Institution (55)	Other (12)
Reward effectiveness	58	–	1	[0.2, 1]	–	–	–
Reward incentive	143	–	–	–	Monetary (102)	Non-monetary Social (23)	Hypothetical (17)
Reward iterations	133	–	1	[0, 1]	–	–	–
Reward probability	124	–	1	[0, 1]	–	–	–
Reward rule	72	–	–	–	Contribution-based (42)	Rank-based (27)	Random (2)
Reward treatment	211	69%	–	–	–	–	–
Sequential punishment	703	–	–	–	Simultaneous (696)	Sequential turn-taking (5)	Sequential leadership-by-example (2)
Sequential reward	133	–	–	–	Simultaneous (131)	Sequential leadership-by-example (1)	Sequential turn-taking (1)
Sequentiality	331	–	–	–	Sequential turn-taking (221)	Simultaneous (102)	Sequential leadership-by-example (8)
Shadow of the future	23	48%	–	–	–	–	–
Show-up fee	89	33%	–	–	–	–	–
Similarity level	26	–	–	–	High (13)	Low (13)	–
Size of unit of decision maker	319	–	1	[1, 12]	–	–	–
Small-scale society	9	–	–	–	Eastern Germany (3)	Western Germany (3)	Other (2)
Social capital	28	–	–	–	–	–	–

Independent Variable	n	Boolean variables	Numerical variables		Categorical variables (most frequent values)		
		TRUE %	*Mdn*	Range	First	Second	Third
Social capital level	4	–	–	–	High (2)	Low (2)	–
State trust	11	–	–	–	–	–	–
State trust level	8	–	–	–	High (3)	Medium (3)	Low (2)
Step return	16	–	5	[0.5, 15]	–	–	–
Step return level	16	–	–	–	Medium (7)	Low (5)	High (4)
Student sample	79	58%	–	–	–	–	–
Subscale	791	31%	–	–	–	–	–
SVO type	433	–	–	–	Prosocial (197)	Proself (148)	Individualist (44)
Symmetric endowment	396	30%	–	–	–	–	–
Symmetric MPCR	61	52%	–	–	–	–	–
Symmetric private account return	6	17%	–	–	–	–	–
Symmetry	233	45%	–	–	–	–	–
Symmetry target	232	–	–	–	Endowment (130)	Payoff matrix (35)	MPCR (29)
Synchrony	16	44%	–	–	–	–	–
Threshold	129	–	26	[0, 140000]	–	–	–
Threshold level	70	–	–	–	High (25)	Low (23)	Medium (22)
Time pressure	87	–	–	–	Time pressure (42)	Time delay (39)	Control (6)
Trial of cooperation	83	–	3	[1, 20]	–	–	–
Trial of cooperation (ordinal)	211	–	–	–	First (98)	Last (96)	Other (17)
Uncertainty level	301	–	–	–	Low (129)	High (126)	Medium (46)
Uncertainty target	306	–	–	–	Resource size (114)	Incentive structure (35)	Threshold (22)
Values (Schwartz)	119	–	–	–	Benevolence (14)	Universalism (14)	Achievement (12)
Vote outcome	28	–	–	–	Yes vote (17)	No vote (11)	–
Vote target	113	–	–	–	Establish (91)	Other (16)	Remove (6)
Watching eyes	7	57%	–	–	–	–	–

Note. n = Frequency of treatments for which a specific independent variable has been annotated, TRUE % = Percentage of values annotated as TRUE for each respective independent variables of type Boolean.

5. Analyses

Meta-analysis. Meta-analysis is implemented through the R package *metafor* (Viechtbauer, 2010), which enables a choice between effect size estimates (*d*, *r*) and fixed- and random-effects models with different estimators. The meta-analysis function *rma.uni()* takes as its input the effect size and associated variance for each effect size. For a regular meta-analysis, the output is an estimate of the overall effect size, its confidence interval, a prediction interval, and estimates of excess heterogeneity.

Users can choose to implement moderators using variables at the observation level (‘variable moderators’), the study level (‘study moderators’), and country level (‘country/region moderators’). Observation-level moderators are defined as the combination of values in both treatments. For example, for studies on punishment, the moderator punishment agent indicates who could enact punishment. When the first treatment involves peer punishment and the second treatment involves institutional punishment, the moderator will take the value ‘peer vs. institution’. When the second treatment does not involve punishment (e.g., a baseline treatment), the moderator takes the value ‘peer vs. NA’. For each categorical moderator, the alphabetically first level is set as the baseline level. The output of a moderated meta-analysis is a regression table showing an estimate of the effect of each moderator on the estimated effect size.

Users can also run multilevel meta-analyses which account for clustering of errors. This uses the function *rma.mv()* in *metafor*. For the purpose of multilevel analysis, sub-studies are considered as belonging to the same study.

Meta-regression. Beyond meta-analyses of effect sizes, CoDa also offers integrative analyses on the rate of cooperation across studies. For this purpose, we compute a standardized proportion of cooperation for each treatment (p_C), either obtained from the

proportion of cooperative choices in dichotomous-choice games (p_{dich}), or from proportion of the total endowment contributed (or resource withdrawn from a common pool) in continuous-choice games.

The proportion of the endowment contributed (p_{cont}) is computed by scaling the mean contribution by the range of the endowment $[E_{LL}, E_{UL}]$. For resource withdrawn from a common pool, we first inverted the mean withdrawals M_w and then scaled it by the choice range $[E_{LL}, E_{UL}]$ to reflect cooperation (p_{cont}) as $\bar{X} = E_{UL} + E_{LL} - M_w$.

Thus, for both continuous-choice games, p_{cont} is computed as

$$p_{cont} = \frac{\bar{X} - E_{LL}}{E_{UL} - E_{LL}}.$$

The proportion of cooperation p_C is then converted to logit, as the effect size, to allow for exploring the heterogeneity among effect sizes (Lipsey & Wilson, 2000) as

$$logit\ of\ p_C = \begin{cases} \log_e\left[\dfrac{p_{dich}}{1 - p_{dich}}\right], & for\ dichotomous\ choices \\ \log_e\left[\dfrac{\bar{X} - E_{LL}}{E_{UL} - \bar{X}}\right], & for\ continuous\ choices \end{cases}$$

To allow comparisons across outcome variables on different scales, we compute the variance v of logit-transformed cooperation (Lipsey & Wilson, 2000) as

$$v = \begin{cases} \dfrac{1}{np_{dich}} + \dfrac{1}{n(1 - p_{dich})}, & for\ dichotomous\ choices \\ \dfrac{s^2}{(\bar{X} - E_{LL})^2} \times \dfrac{1}{n(1 - p_{cont})^2}, & for\ continuous\ choices \end{cases}$$

where s is the standard deviation.

Meta-regression is based on the same observation-centric search and selection functions as meta-analysis. To accommodate analyses of treatments, the data are filtered in several ways. First, any selected moderators are applied, retaining only observations that include the selected

moderator. Subsequently, if for any study there are multiple variables with observations that fit the selected criteria, only one variable is retained. To ensure reproducibility, CoDa always retains the variable with the first-ranked variable ID. Subsequently, from these variables only the treatments that fit the selected treatment criteria are retained. Finally, for within-subjects comparisons, if multiple treatments fit the criteria, only the first-ranked treatment (by treatment ID) is retained. This procedure ensures that there are no duplications; i.e., participants are never included twice in an analysis. Meta-regression is implemented using *metafor*.

7. The CoDa Research Application and Platform

Figure S1

Overview of Selected Data

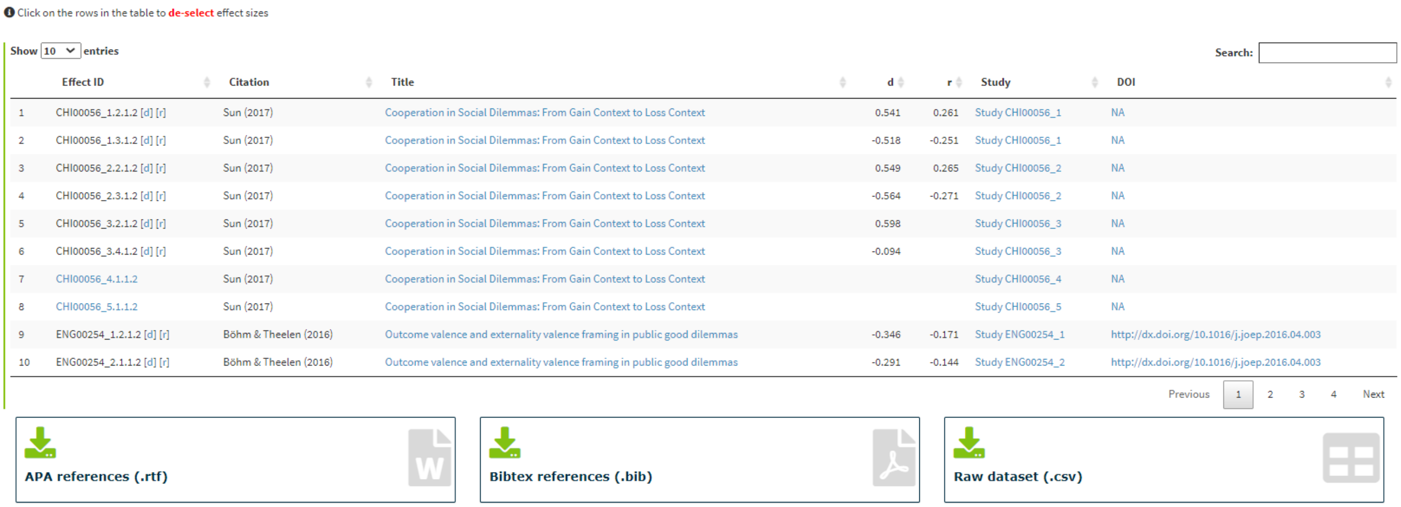
Click on the rows in the table to de-select effect sizes

Show 10 entries Search:

	Effect ID	Citation	Title	d	r	Study	DOI
1	CHI00056_1.2.1.2 [d] [r]	Sun (2017)	Cooperation in Social Dilemmas: From Gain Context to Loss Context	0.541	0.261	Study CHI00056_1	NA
2	CHI00056_1.3.1.2 [d] [r]	Sun (2017)	Cooperation in Social Dilemmas: From Gain Context to Loss Context	-0.518	-0.251	Study CHI00056_1	NA
3	CHI00056_2.2.1.2 [d] [r]	Sun (2017)	Cooperation in Social Dilemmas: From Gain Context to Loss Context	0.549	0.265	Study CHI00056_2	NA
4	CHI00056_2.3.1.2 [d] [r]	Sun (2017)	Cooperation in Social Dilemmas: From Gain Context to Loss Context	-0.564	-0.271	Study CHI00056_2	NA
5	CHI00056_3.2.1.2 [d] [r]	Sun (2017)	Cooperation in Social Dilemmas: From Gain Context to Loss Context	0.598		Study CHI00056_3	NA
6	CHI00056_3.4.1.2 [d] [r]	Sun (2017)	Cooperation in Social Dilemmas: From Gain Context to Loss Context	-0.094		Study CHI00056_3	NA
7	CHI00056_4.1.1.2	Sun (2017)	Cooperation in Social Dilemmas: From Gain Context to Loss Context			Study CHI00056_4	NA
8	CHI00056_5.1.1.2	Sun (2017)	Cooperation in Social Dilemmas: From Gain Context to Loss Context			Study CHI00056_5	NA
9	ENG00254_1.2.1.2 [d] [r]	Böhm & Theelen (2016)	Outcome valence and externality valence framing in public good dilemmas	-0.346	-0.171	Study ENG00254_1	http://dx.doi.org/10.1016/j.joep.2016.04.003
10	ENG00254_2.1.1.2 [d] [r]	Böhm & Theelen (2016)	Outcome valence and externality valence framing in public good dilemmas	-0.291	-0.144	Study ENG00254_2	http://dx.doi.org/10.1016/j.joep.2016.04.003

Previous 1 2 3 4 Next

APA references (.rtf)
Bibtex references (.bib)
Raw dataset (.csv)

Note. Partial screenshot of the frontpage of the CoDa platform. The table documents the result of the selection, allowing the download of references and raw data of the selected studies.

Figure S2

Example of Meta-Analytic Output Reported on the CoDa Platform

The following interpretation is derived from the selections that you have made:

Meta-analysis comparing Treatment 1 (Gain-Loss frame: Gain) against Treatment 2 (Gain-Loss frame: Loss) on cooperation. A positive effect size indicates that cooperation is higher in Treatment 1 (Gain-Loss frame: Gain) than in Treatment 2 (Gain-Loss frame: Loss).

Show 10 entries Search:

	Overall effect size					Heterogeneity estimates		
Effect	k	d	95% CI	Z	p	95% PI	T^2	I^2
Gain-Loss frame: Gain vs. Gain-Loss frame: Loss	20	0.052	[-0.108, 0.212]	0.636	0.525	[-0.547, 0.651]	0.087	76.86

Previous 1 Next

Note. Partial screenshot of the "meta-analytic models" section of the CoDa platform. The table reports a meta-analysis for studies comparing treatments with gain vs. loss framing.

k = number of effect sizes; CI = Confidence Interval; PI = Prediction Interval.

Figure S3

Violin Plot

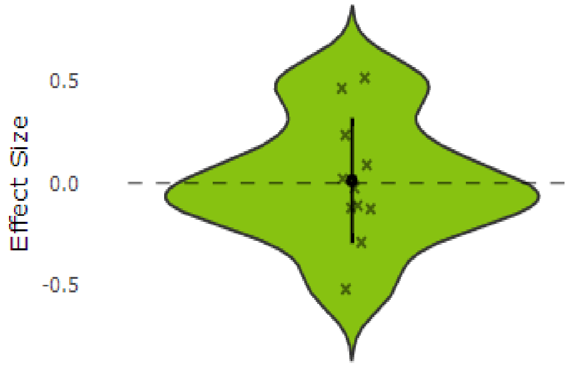

Note. Partial screenshot of the "visualization" section of the CoDa platform. The plot displays the distribution of effect sizes.

Figure S4

Power Analysis

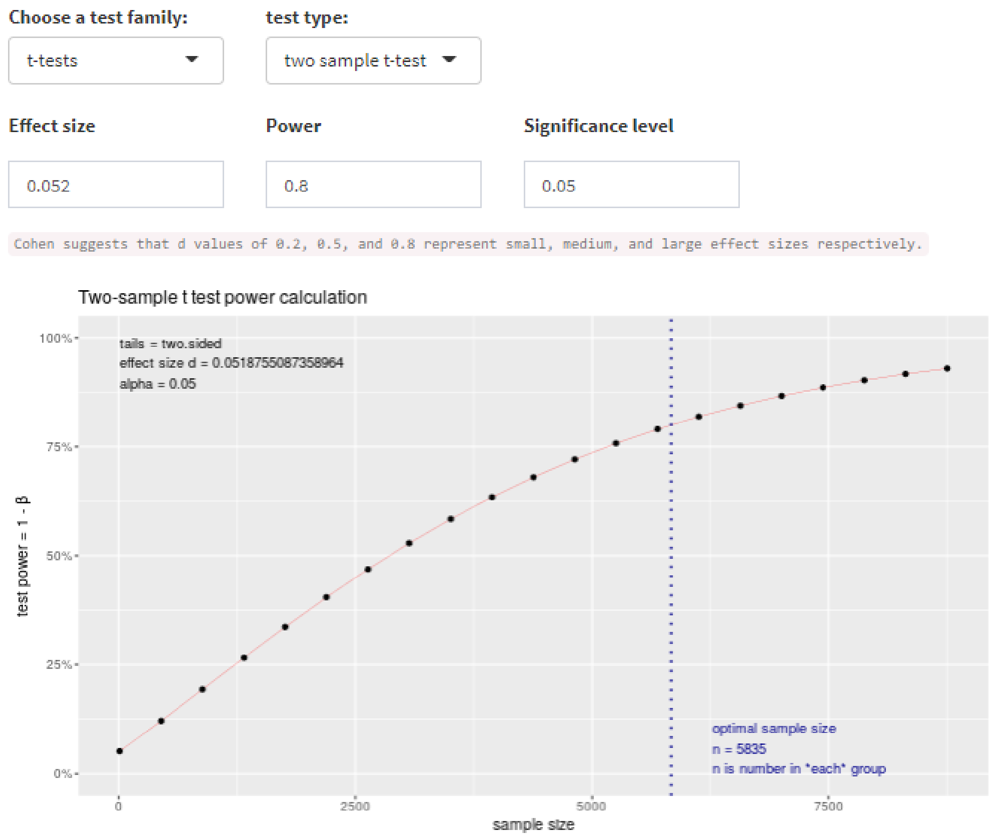

Note. Partial screenshot of the “power analysis” section of the CoDa platform. This feature performs statistical power analysis and calculates the optimal sample size to detect the effect under investigation.

8. References

Atkinson, K. M., Koenka, A. C., Sanchez, C. E., Moshontz, H., & Cooper, H. (2015). Reporting standards for literature searches and report inclusion criteria: Making research syntheses more transparent and easy to replicate. *Research Synthesis Methods*, *6*(1), 87–95. https://doi.org/10.1002/jrsm.1127

Balliet, D. (2010). Communication and cooperation in social dilemmas: A meta-analytic review. *Journal of Conflict Resolution*, *54*(1), 39–57. https://doi.org/10.1177/0022002709352443

Balliet, D., Li, N. P., Macfarlan, S. J., & Van Vugt, M. (2011). Sex differences in cooperation: A meta-analytic review of social dilemmas. *Psychological Bulletin*, *137*(6), 881–909. https://doi.org/10.1037/a0025354

Balliet, D., Parks, C., & Joireman, J. (2009). Social value orientation and cooperation in social dilemmas: A meta-analysis. *Group Processes & Intergroup Relations*, *12*(4), 533–547. https://doi.org/10.1177/1368430209105040

Balliet, D., & van Lange, P. A. M. (2013). Trust, punishment, and cooperation across 18 societies: A meta-analysis. *Perspectives on Psychological Science*, *8*(4), 363–379. https://doi.org/10.1177/1745691613488533

Balliet, D., & Van Lange, P. A. M. (2013). Trust, conflict, and cooperation: A meta-analysis. *Psychological Bulletin*, *139*(5), 1090–1112. https://doi.org/10.1037/a0030939

Balliet, D., Wu, J., & De Dreu, C. K. W. (2014). Ingroup favoritism in cooperation: A meta-analysis. *Psychological Bulletin*, *140*(6), 1556–1581. https://doi.org/10.1037/a0037737

Biel, A., Eek, D., Gärling, T., & Gustafsson, M. (2008). *New issues and paradigms in research on social dilemmas*. Springer. https://doi.org/10.1007/978-0-387-72596-3

Borenstein, M., Hedges, L. V., Higgins, J. P. T., & Rothstein, H. R. (2009). Introduction to meta-analysis. In *Introduction to Meta-Analysis*. John Wiley and Sons. https://doi.org/10.1002/9780470743386

Bornstein, G. (2003). Intergroup conflict: individual, group, and collective interests. *Personality and Social Psychology Review : An Official Journal of the Society for Personality and Social Psychology, Inc*, *7*(2), 129–145. https://doi.org/10.1207/S15327957PSPR0702_129-145

Bouwmeester, S., Verkoeijen, P. P. J. L., Aczel, B., Barbosa, F., Bègue, L., Brañas-Garza, P., Chmura, T. G. H., Cornelissen, G., Døssing, F. S., Espín, A. M., Evans, A. M., Ferreira-Santos, F., Fiedler, S., Flegr, J., Ghaffari, M., Glöckner, A., Goeschl, T., Guo, L., Hauser, O. P., … Wollbrant, C. E. (2017). Registered replication report: Rand, Greene, and Nowak (2012). *Perspectives on Psychological Science*, *12*(3), 527–542. https://doi.org/10.1177/1745691617693624

Chaudhuri, A. (2011). Sustaining cooperation in laboratory public goods experiments: A selective survey of the literature. *Experimental Economics*, *14*(1), 47–83. https://doi.org/10.1007/s10683-010-9257-1

Cohen, J. (1988). *Statistical power analysis for the behavioral sciences*. Routledge Academic.

Dawes, R. M. (1980). Social dilemmas. *Annual Review of Psychology*, *31*(1), 169–193. https://doi.org/10.1146/annurev.ps.31.020180.001125

Fiala, L., & Suetens, S. (2017). Transparency and cooperation in repeated dilemma games: a meta study. *Experimental Economics*, *20*(4), 755–771. https://doi.org/10.1007/s10683-017-9517-4

Foddy, M. (2013). *Resolving social dilemmas*. Psychology Press. https://doi.org/10.4324/9780203776001

Hayes, A. F., & Krippendorff, K. (2007). Answering the call for a standard reliability measure for coding data. *Communication Methods and Measures*, *1*(1), 77–89. https://doi.org/10.1080/19312450709336664

Henrich, J., Boyd, R., Bowles, S., Camerer, C. F., Fehr, E., & Gintis, H. (2005). *Foundations of human sociality: Economic experiments and ethnographic evidence from fifteen small-scale societies*. Oxford University Press. https://doi.org/10.1093/0199262055.001.0001

Herrmann, B., Thöni, C., & Gächter, S. (2008). Antisocial punishment across societies. *Science, 319*(5868), 1362–1367. https://doi.org/10.1126/science.1153808

Jones, A. P., Johnson, L. A., Butler, M. C., & Main, D. S. (1983). Apples and oranges: An empirical comparison of commonly used indices of interrater agreement. *Academy of Management Journal*, *26*(3), 507–519. https://doi.org/10.5465/256262

Komorita, S. S., Parks, C. D. (1995). *Social dilemmas*. Brown & Benchmark.

Krippendorff, K. (2011). Computing Krippendorff's Alpha-Reliability. In *Departmental Papers (ASC)*. https://repository.upenn.edu/asc_papers/43

Liebrand, W. B. G., Messick, D. M. (1996). Frontiers in social dilemmas research. In *Frontiers in Social Dilemmas Research*. Springer Berlin Heidelberg. https://doi.org/10.1007/978-3-642-85261-9

Lipsey, M. W., & Wilson, D. B. (2000). *Practical meta-analysis*. Sage.

Lüdecke, D. (2019). *Esc: effect size computation for meta analysis (version 0.5.1)*. https://doi.org/10.5281/zenodo.1249218

Mengel, F. (2018). Risk and temptation: A meta-study on prisoner's dilemma games. *The Economic Journal*, *128*(616), 3182–3209. https://doi.org/10.1111/ecoj.12548

Page, M. J., McKenzie, J. E., Bossuyt, P. M., Boutron, I., Hoffmann, T. C., Mulrow, C. D., Shamseer, L., Tetzlaff, J. M., Akl, E. A., Brennan, S. E., Chou, R., Glanville, J., Grimshaw, J. M., Hróbjartsson, A., Lalu, M. M., Li, T., Loder, E. W., Mayo-Wilson, E., McDonald, S., … Moher, D. (2021). The PRISMA 2020 statement: An updated guideline for reporting systematic reviews. *BMJ, 372*, n71. https://doi.org/10.1136/bmj.n71

Parks, C. D. (2000). Testing various types of cooperation rewards in social dilemmas. *Group Processes & Intergroup Relations, 3*(4), 339–350. https://doi.org/10.1177/1368430200003004001

Pletzer, J. L., Balliet, D., Joireman, J., Kuhlman, D. M., Voelpel, S. C., & Van Lange, P. A. M. (2018).

Social value orientation, expectations, and cooperation in social dilemmas: A meta-analysis. *European Journal of Personality*, *32*(1), 62–83. https://doi.org/10.1002/per.2139

Pruitt, D. G., & Kimmel, M. J. (1977). Twenty years of experimental gaming: Critique,synthesis, and suggestions for the future. *Annual Review of Psychology*, *28*(1), 363–392. https://doi.org/10.1146/annurev.ps.28.020177.002051

Rand, D. G. (2016). Cooperation, fast and slow: Meta-analytic evidence for a theory of social heuristics and self-interested deliberation. *Psychological Science*, *27*(9), 1192–1206. https://doi.org/10.1177/0956797616654455

Rapoport, A., Chammah, A. M., & Orwant, C. J. (1965). *Prisoner's dilemma: A study in conflict and cooperation* (Vol. 165). University of Michigan press.

Sally, D. (1995). Conversation and cooperation in social dilemmas: A meta-analysis of experiments from 1958 to 1992. *Rationality and Society*, *7*, 58–92. https://doi.org/10.1177/1043463195007001004

Suleiman, R., Budescu, D. V, Fischer, I., & Messick, D. M. (2004). *Contemporary psychological research on social dilemmas*. Cambridge University Press.

Thielmann, I., Spadaro, G., & Balliet, D. (2020). Personality and prosocial behavior: A theoretical framework and meta-analysis. *Psychological Bulletin*, *146*(1), 30–90. https://doi.org/10.1037/bul0000217

Van Dijk, E., & Wilke, H. (1994). Asymmetry of Wealth and Public Good Provision. *Social Psychology Quarterly*, *57*(4), 352–359. https://doi.org/10.2307/2787161

Variance of Cohen's d for within subjects designs. (2017). https://stats.stackexchange.com/questions/256053/variance-of-cohens-d-for-within-subjects-designs/256205#256205

Viechtbauer, W. (2010). Conducting meta-analyses in R with the metafor. *Journal of Statistical Software*, *36*(3), 1–48. https://doi.org/10.18637/jss.v036.i03

Westfall, J. (2016). *Five different "Cohen's d" statistics for within-subject designs*. http://jakewestfall.org/blog/index.php/2016/03/25/five-different-cohens-d-statistics-for-within-subject-designs/

Wrightsman, L. S., O'Connor, J., & Baker, N. J. (1972). *Cooperation and competition: Readings on mixed-motive games*. BrooksCole Pub. Co.
